# Innovative research on predicting skin flaps necrosis through quantification of the USP4/TNFAIP2 regulated ‘M1 macrophages-Tregs axis’ via skin barrier monitoring technology

**DOI:** 10.1093/burnst/tkag016

**Published:** 2026-02-27

**Authors:** Xuanlong Zhang, Xiaoqiong Jiang, Shenjie Song, Kaiyi Du, Hongxin Wang, Zihan Zhang, Yu Wang, Xiangwei Ling, Qingfeng Li

**Affiliations:** Department of Plastic and Reconstructive Surgery, Shanghai Ninth People's Hospital, Shanghai Jiao Tong University School of Medicine, 639 Zhizaoju Road, Huangpu District, Shanghai 200011, China; Shanghai Institute for Plastic and Reconstructive Surgery, 1908 Gaoke West Road, Pudong New Area, Shanghai 200011, China; School of Nursing, Wenzhou Medical University, Chashan University Town, Ouhai District, Wenzhou 325035, Zhejiang, China; School of Clinical Medicine, North Sichuan Medical College, 234 Fujiang Road, Shunqing District, Nanchong 637000, Sichuan, China; School of Pharmaceutical Sciences, Wenzhou Medical University, Chashan University Town, Ouhai District, Wenzhou 325035, Zhejiang, China; School of Nursing, Wenzhou Medical University, Chashan University Town, Ouhai District, Wenzhou 325035, Zhejiang, China; School of Nursing, Wenzhou Medical University, Chashan University Town, Ouhai District, Wenzhou 325035, Zhejiang, China; Department of Medical Engineering, The First Affiliated Hospital of Wenzhou Medical University, Ouhai District, Wenzhou 325000, Zhejiang, China; Burn Department, The First Affiliated Hospital of Wenzhou Medical University, Ouhai District, Wenzhou 325000, Zhejiang, China; Department of Plastic and Reconstructive Surgery, Shanghai Ninth People's Hospital, Shanghai Jiao Tong University School of Medicine, 639 Zhizaoju Road, Huangpu District, Shanghai 200011, China; Shanghai Institute for Plastic and Reconstructive Surgery, 1908 Gaoke West Road, Pudong New Area, Shanghai 200011, China

**Keywords:** Skin barrier, Skin flaps, Macrophages, Tregs, Ferroptosis, USP4, TNFAIP2

## Abstract

**Background:**

Current methods used for assessing random-pattern skin flaps (RPSFs) viability are invasive and subjective. Therefore, this study aimed to establish an accurate and non-invasive method for predicting RPSFs prognosis using skin barrier monitoring technology.

**Methods:**

Gene ontology (GO) analysis was employed to identify the potential pathways associated with RPSFs necrosis. Skin barrier monitoring technology, immunohistochemistry (IHC), immunofluorescence (IF), and western blotting (WB) were employed to assess skin barrier integrity, quantify ferroptosis level, and evaluate the recruitment of M1 macrophages and regulatory T cells (Tregs). Clinical samples were also included to validate the accuracy and efficacy of skin barrier monitoring technology in predicting RPSFs necrosis.

**Results:**

The necrotic process in RPSFs was characterized by increased infiltration of M1 macrophages and Tregs, accompanied by activated ferroptosis and dysfunction of skin barrier. Notably, Tregs infiltration significantly reduced the necrotic area and restored skin barrier integrity of RPSFs by inhibiting ferroptosis and polarization of M1 macrophages. Mechanistically, USP4 regulated the interaction of M1 macrophages and Tregs (M1 macrophages-Tregs axis) via stabilization of TNFAIP2. Analysis of clinical samples revealed that skin barrier monitoring technology could serve as predictive strategy for RPSFs necrosis.

**Conclusions:**

This study demonstrated that the USP4/TNFAIP2 pathway would regulate skin barrier function and ferroptosis via ‘M1 macrophages-Tregs axis’ in RPSFs. And the skin barrier monitoring technology could serve as reliable method for RPSFs necrosis prediction.

## Graphical abstract

**Graphical abstract:**
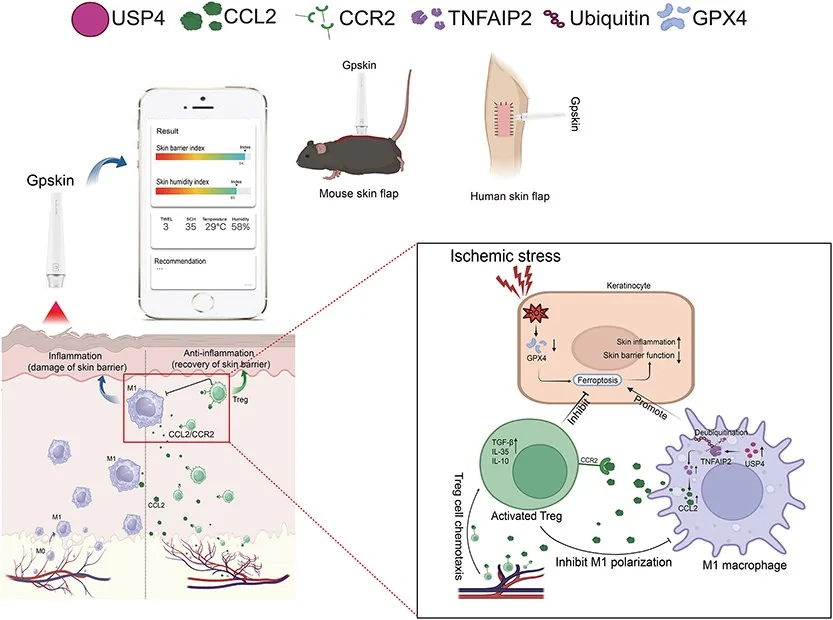

## Highlights

- Non-invasive skin barrier monitoring technology provides an accurate strategy for predicting necrosis of random-pattern skin flaps (RPSFs).
- Relative transepidermal water loss and relative stratum corneum hydration serve as reliable predictive markers for necrosis in RPSFs.
- USP4 stabilizes TNFAIP2 through deubiquitination to regulate skin barrier function of RPSFs via M1 macrophages–Tregs axis.

## Background

The blood supply of random-pattern skin flaps (RPSFs) is derived principally from the subcutaneous vasculature rather than axial vessels [1, 2]. However, RPSFs exhibit pronounced susceptibility to ischemia and hypoxia, rendering postoperative viability assessment critically important. Current clinical evaluation methods—including vascular perfusion assessment, visual inspection of RPSFs appearance, edema evaluation, and biopsy—are widely employed. Nevertheless, the inherent subjectivity and invasiveness of these traditional strategies represent notable limitations. To overcome this constraint, we sought to establish an objective, non-invasive methodology for predicting RPSFs prognosis, thereby furnishing clinicians with robust diagnostic evidence to inform personalized therapeutic strategies.

The skin barrier, primarily composed of keratinocytes, functions to limit water loss and maintain cutaneous hydration [3, 4]. Transepidermal water loss (TEWL) and stratum corneum hydration (SCH) have emerged as objective parameters for assessing skin barrier integrity [5, 6], with diminished skin water content reflecting greater barrier impairment. Ferroptosis, an iron-dependent modality of regulated cell death, demonstrates close association with skin barrier disruption and M1 macrophage polarization [7, 8]. The present study revealed that ischemia/hypoxia-induced ferroptosis compromised skin barrier function prior to the appearance of evident necrosis in mice RPSFs. A parallel phenomenon was observed in human RPSFs, wherein skin barrier function progressively deteriorated before necrosis became evident, suggesting that skin barrier indexes may serve as early predictive indicators for RPSFs necrosis.

Excessive infiltration of M1 macrophages exacerbates skin barrier damage in RPSFs through inflammatory responses [9]. Tregs not only suppress the activation of M1 macrophages by secreting cytokines such as interleukin-35 (IL-35) but also mitigate keratinocyte injury and skin barrier impairment [10, 11]. In addition, infiltration of Tregs may therefore promote the survival of RPSFs [12]. Consequently, modulating the crosstalk between M1 macrophages and Tregs represents a promising therapeutic strategy to enhance RPSFs survival. Moreover, the concept of an ‘M1 macrophages-Tregs axis’ in the context of the RPSFs skin barrier was proposed for the first time and elucidating this crosstalk would not only advance the understanding of immune regulation in skin flap necrosis but also offer novel theoretical frameworks for RPSFs recovery.

Ubiquitin-specific protease 4 (USP4) is a deubiquitinating enzyme that removes ubiquitin from protein substrates, thereby modulating post-translational modifications and cellular functions [13]. Emerging evidence has demonstrated that USP4 is intricately involved in multiple ischemic and inflammatory signaling pathways [14, 15]. The present study revealed that USP4 expression was upregulated in the necrotic area of RPSFs, suggesting a potential role for USP4-mediated inflammatory regulation in skin flap. However, the mechanisms by which USP4 influences the immune functions of macrophages and Tregs remain poorly defined, and its involvement in skin regeneration and barrier reconstruction has been scarcely reported. Mass spectrometry data indicated that TNFAIP2 may serve as a potential downstream target of USP4. TNFAIP2 has been established as an important mediator of inflammatory and immune responses [16]. Nevertheless, the regulatory mechanism of TNFAIP2 in the skin barrier of ischemic skin flap has yet to be investigated. Therefore, we hypothesized that the USP4/TNFAIP2 signaling axis modulates the ‘M1 macrophages-Tregs axis’, thereby affecting skin barrier integrity and necrosis in RPSFs through the regulation of ferroptosis and inflammation. This study was designed to test this hypothesis and to evaluate the potential of skin barrier indexes as early predictive biomarkers for RPSFs necrosis.

## Methods

### Experimental animals

The experimental animals were 6- to 8-week-old male C57BL/6 mice. The mice were weighed on average 25–30 grams and were housed in a standard environment (temperature: 21–25°C, humidity: 50%–60%, 12-hour light/dark cycle). The animals had free access to food and water. The housing of the experimental animals was approved by the Animal Committee of Wenzhou Medical University (XMSSQ2023–0005). First, the mice were anesthetized by intraperitoneal injection of pentobarbital sodium solution at a dose of 50 mg/kg. After the mice were anesthetized, the hair on the back of the mice was completely removed. Subsequently, a rectangular RPSFs with size of 1.5 cm × 4.5 cm was designed on the dorsum of mice. After the skin flap was fully surgically dissected, all visible blood vessels under the skin flap were carefully removed, and then the mice skin flap was sutured with 4–0 non-absorbable silk thread. In addition, vectors were respectively used to regulate the expression of downregulate USP4 and upregulateTNFAIP2 after skin flap surgeries. Subsequently, the mice were divided into the following groups: PBS group, Erastin group, Tregs group, shNC group, shUSP4 group, shUSP4 + TNFAIP2 group, CL group, RS504393 group, and Peptide P60 group. Furthermore, vivo-jetPEI polyplus was employed for *in vivo* delivery of USP4 plasmid and si-TNFAIP2 sequences to respectively upregulate USP4 or downregulate TNFAIP2 expression. According to the manufacturer’s instructions, the nucleic acids were complexed with the vivo-jetPEI polyplus reagent and administered via daily subcutaneous injection from Day 1 to Day 7.

The percentage of the necrotic area of mice skin flap: the anesthetized mice were placed in a quiet environment, and the skin flap images were captured by a digital camera. The skin flap images were processed and analyzed using ImageJ software. The necrotic area and the total area of the mice skin flap were respectively calculated. Percentage of the necrotic area of mice skin flap = necrotic area of the skin flap/total area of the skin flap × 100%. Measurement of the skin moisture index (skin barrier index): The mice skin barrier of skin flap was detected using a skin barrier detection device (Gpskin). First, the skin flap was divided into three equal-sized sections: Area I, Area II, and Area III. After anesthesia of the mice, the Gpskin device was gently placed on the three different sections of the skin flap, and then the device was started for measurement. Finally, the TEWL, SCH, and humidity indexes were manifested on the screen. The relative TEWL (RT), relative SCH (RS), and relative humidity (RH) indexes were calculated as following: RT/RS/RH of the skin flap = TEWL/SCH/Humidity index of the skin flap—TEWL/SCH/Humidity index of the uninjured skin. In order to demonstrate the inter-observer reliability and temporal stability of Gpskin, two independent operators were employed to perform skin barrier assessments of the mice RPSFs on D1, D3, and D7. Measurements were taken three times daily at 8:00 a.m., 8:15 a.m., and 8:30 a.m. The quantification of RT and RS was performed using the same method as described above. The average values obtained from three measurements of each mouse were statistically analyzed. The skin samples from Area II were used for WB, quantitative real-time polymerase chain reaction (qRT-PCR) and immunofluorescence (IF) analysis, etc.

### Clinical patients inclusion criteria

This clinical study was initiated by The First Affiliated Hospital of Wenzhou Medical University and The Second Affiliated Hospital of Wenzhou Medical University. The patients who underwent RPSFs transplantation were recruited from February 2025 to August 2025. Patients with hypertension, diabetes, chronic vascular diseases were excluded. This study was carried out in strict accordance with the ethical standards stipulated in the declaration of Helsinki II, and this study was approved by the Ethics Committee of the Affiliated Research of Wenzhou Medical University (no.: KY2023-294, 2023-SCYJ-006, and KY2025-R392). All the participants or their legal guardians were fully informed of the relevant content of this experiment. First, we conducted a multicenter clinical trial which allowed us to examine the correlation between skin barrier parameters and skin flap necrosis, and to calculate the predictive thresholds.

### Prospective clinical trial

Another validation cohort study was conducted to analyze the utility, sensitivity, and specificity of RT and RS displayed by Gpskin. In this prospective clinical trial, patients undergoing RPSFs surgeries were randomly assigned to two groups (Group A and Group B). The patients’ characteristics were listed in Table 2 of supplementary data. In both groups, Gpskin was used to monitor and record the RT and RS indexes from postoperative Day 1 to Day 7. If the patients’ skin flaps were diagnosed with possible necrosis postoperatively, the following treatment should be immediately administered. The treatment include heparin sodium moist compress, position adjustment, infrared therapy, tension-reducing therapy, and blood circulation medications. However, the diagnostic methods and the timing of treatment were different in the two groups. In Group A, Gpskin was used to diagnose the necrotic possibility of RPSFs, and the patients received immediate clinical interventions when the skin barrier indexes recorded by Gpskin was altered (RT > 3.5, RS < −2.5). In Group B, traditional diagnostic methods (vascular perfusion assessment, visual inspection of skin flap appearance, and edema evaluation) were used to detect necrotic possibility of RPSFs, and the timing of postoperative interventions depended primarily on the patients’ symptoms and the clinicians’ observations. Finally, statistical analysis was performed on the skin flap survival rate and skin barrier indexes of these patients to validate the clinical advantages of Gpskin.

### Western blotting

The skin flap in Area II and human skin samples were both weighed, and 800 μl of RIPA lysis buffer (Beyotime) was added for every 100 mg of tissue, and protease and phosphatase inhibitors (Sigma-Aldrich) were also added. The protein extraction steps for macrophages were basically the same as those for skin tissue. The protein samples were loaded onto a sodium dodecyl sulfate polyacrylamide gel electrophoresis (SDS-PAGE) gel and then transferred to polyvinylidene fluoride (PVDF) membranes. The PVDF membranes were incubated in a blocking solution containing 5% skimmed milk for 2 hours. The membranes were washed thoroughly three times with TBST buffer, and finally incubated with primary and secondary antibodies (supplementary data, Table antibodies list). The PVDF membranes were incubated with the primary antibodies at 4°C overnight. Following the incubation, the membrane was washed three times with TBST buffer. Subsequently, the membrane was incubated with the secondary antibodies at room temperature for 2 hours. Finally, the membrane was washed three times with TBST. The immunoreactive bands were then visualized using an ECL immunoblotting (IB) detection kit. The image of WB was captured by ChemiDoc™ XRS + imaging system (Bio-Rad). The WB images were first quantified using Image J software to obtain the grayscale density values for each protein. The grayscale density value of the targeted protein was then normalized to that of the GAPDH to generate the relative ratio. This ratio was subsequently subjected to normalization against the control group, ultimately yielding the final statistical graphs. Statistical methods such as the *t*-test, one-way analysis of variance (ANOVA), Tukey’s honestly significant difference (HSD) test and Dunnett’s T3 test were used (the specific statistical methods were described in statistical analysis section). In addition, ELISA experiments were used to detect the concentration of IL-35 in mice skin flap.

### Quantitative real-time polymerase chain reaction assay

The isolation and extraction of total RNA from cells and skin tissues were completed by Trizol reagent. The extracted RNA was purified, and its concentration was determined using a NanoDrop 2000 (Thermo Scientific). The total RNA was placed in a GeneAmp PCR system (Applied Biosystems), and reverse transcription was carried out using HiScript III RT SuperMix (+gDNA wiper) (Vazyme). Subsequently, an amplification experiment was conducted using Taq Pro Universal SYBR qPCR Master Mix (Vazyme). The primers were shown in supplementary data (Supplementary data, Table PCR primers list).

### Immunofluorescence and immunohistochemistry assay

The mice skin flap of Area II were harvested. After extruding the excess red blood cells with PBS solution, the tissues were fixed with 4% paraformaldehyde. Then the skin tissues were dehydrated, embedded in paraffin. The tissue sections were incubated overnight with primary antibodies (supplementary data, Table antibodies list) at 4°C. Following incubation, the tissue sections were washed three times with PBS buffer. Subsequently, the sections were incubated with a fluorescently-labeled secondary antibody at room temperature for 2 hours. After incubation with secondary antibodies. The nuclei were stained with 4′,6-diamidino-2-phenylindole (DAPI) (Beyotime). Finally the images of sections were captured by microscope. The images were also analyzed using Image J software to quantify the number of positive cells and the mean fluorescence intensity of the targeted protein. Statistical methods such as the *t*-test, one-way ANOVA, Tukey’s HSD test, and Dunnett’s T3 test were used (the specific statistical methods were described in statistical analysis section). The procedure of immunohistochemistry (IHC) was the partially same as above. And the antibodies used was listed in supplementary data.

HaCat were seeded on sterile slices and placed in a 6-well culture dish. The original medium of the treated cells was removed. First, the keratinocyte slices were fixed in 4% formaldehyde solution for 20 minutes. The cells samples were permeabilized with 0.2% Triton X-100 solution for 30 minutes. The cells were blocked with 3% BSA blocking solution for antigen. The specific primary antibody anti-GPX4 (1:200, Proteintech, 67763-1-Ig) was used to incubate overnight at 4°C. The nuclei were stained with DAPI staining agent (Beyotime).

### Hematoxylin and eosin staining

The sections were successively immersed in xylene to remove paraffin, and then hydrated through a series of graded ethanol solutions. The sections were stained in hematoxylin staining solution for 5 to 15 minutes to stain the cells nuclei. After that, the sections were washed with tap water, and then differentiated in 1% hydrochloric acid ethanol solution for 10 seconds. Subsequently, the sections were washed with water for 10 to 15 minutes. The sections were stained in eosin staining solution for 30 seconds to 5 minutes to stain the cytoplasm. The sections were dehydrated successively and then transparentized in xylene. Finally, coverslips were placed on the sections.

### Dihydroethidium staining

The HaCat cells pretreated with tert-butyl hydroperoxide (TBHP) were co-incubated with the supernatant of Tregs, and stained with dihydroethidium (DHE) solution. After the staining was completed, the cells were thoroughly washed with PBS buffer, and then the cells nuclei were stained with Hoechst staining agent (Beyotime).

### Cells culture

The spleens, inguinal, and axillary lymph nodes of healthy male C57BL/6 mice were harvested. After thorough grinding these organs with a syringe, the tissue residues were filtered out and a single-cell suspension was prepared. CD4/CD25 Tregs were isolated using a Tregs isolation kit (Miltenyi). The splenic cells suspension was firstly incubated with a non-CD4 depletion cocktail to remove non-CD4-positive cells. The cells suspension was directly applied to a MACS Column. During this process, the CD4/CD25 positive Tregs, which were magnetically labeled with CD25 microbeads, were specifically retained and enriched within the column, while unlabeled cells were efficiently washed away. The isolated cells were cultured using a Tregs culture medium (Sunncell). The isolated Tregs were seeded into culture flasks and monitored daily for proliferation. Once the cells reached approximately 80–90% confluence, they were harvested by centrifugation and subsequently subcultured. Further experiments were conducted when a sufficient number of Tregs had been expanded. TBHP was used to co-culture with Tregs to mimic the oxidative condition in skin flap. After the culture, the cells culture supernatant was also collected for the next experiments. Human adult cutaneous keratinocytes (HaCat) and macrophages (RAW 264.7) were cultured in a complete medium containing 10% fasting blood sugar and 1% penicillin–streptomycin solution (Gibco), and cultured under the conditions of 37°C and 5% CO_2_.

HaCat were pretreated with TBHP at a concentration of 100 μmol/ml for 24 hours to simulate oxidative stress injury caused by ischemia and hypoxia. Subsequently, the TBHP-Tregs supernatant was co-incubated with the treated HaCat for another 24 hours, followed by subsequent DHE, IF, and flow cytometry (FCM) experiments. RAW 264.7 were also stimulated with TBHP for 24 hours. Then the activated RAW 264.7 cells were co-incubated with the Tregs supernatant for another 24 hours. The cells suspension was incubated with F4/80-PB450 antibody and cluster of differentiation 86 (CD86)-APC antibody at 4°C for 30 minutes. Finally, detection was carried out using an FCM instrument.

### Transfection and co-immunoprecipitation experiment

Flag-tagged USP4, Flag-tagged USP4^C311A^, Myc-tagged-TNFAIP2, and HA-tagged-Ub plasmids were transfected into RAW 264.7 cells. After 48 hours of cells culture, macrophages were lysed thoroughly with RIPA lysis buffer containing protease inhibitors (Beyotime). Subsequently, the lysate was co-incubated with primary antibody and protein A/G-agarose beads. Then, the bound proteins were eluted by boiling, and WB analysis was performed using SDS-PAGE. In addition, viral transfection technology was used to regulate the expression of proteins *in vivo* and *in vitro*. shUSP4 virus and TNFAIP2 virus were used to reduce the USP4 and TNFAIP2 proteins in RPSFs of mice or RAW 264.7 cells.

### Statistical analysis

All experimental data are presented as the mean ± standard error of the mean (SEM). The sample size for each group is denoted by 'n' , representing the number of independent biological or experimental replicates. Statistical analysis was performed using SPSS software (Chicago, Illinois, USA). For comparisons between two groups, paired or independent samples *t*-tests were used as appropriate. For comparisons involving three or more groups, data were first analyzed by one-way ANOVA. To control the Type I error rate in subsequent multiple comparisons, appropriate post-hoc tests were selected based on the experimental design and variance homogeneity (assessed by Levene's test). For all pairwise comparisons among groups, if variances were homogeneous, Tukey's HSD test was applied; if variances were heterogeneous, Dunnett's T3 test was used. For comparisons where all treatment groups were to be compared against a single control group, Dunnett' s test was employed directly following ANOVA. *P* value <0.05 was considered statistically significant. ^*^ stands for *P* < 0.05, ^*^ stands for *P* < 0.01, ^***^ stands for *P* <0.001, ^****^ stands for *P* < 0.0001 and ns stands for no statistical significance.

## Results

### Random-pattern skin flaps necrosis was correlated with skin barrier dysfunction

**Figure 1:**
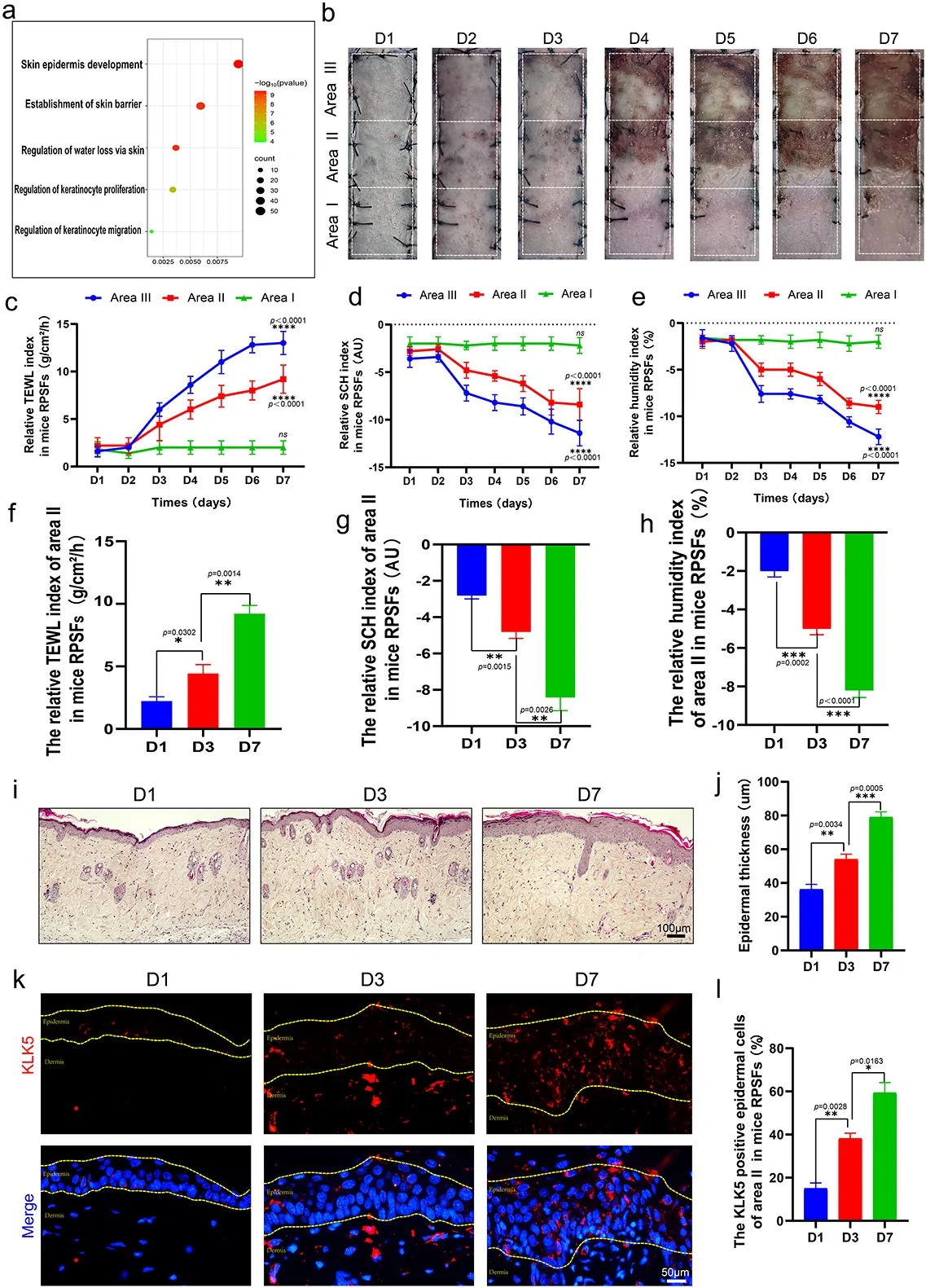
RPSFs necrosis was correlated with skin barrier dysfunction. (**a**) Representative GO pathway analysis of differentially expressed genes associated with skin barrier function, water loss via skin, and epidermis development in mice RPSFs. (**b**) Representative photographs of mice RPSFs from postoperative Day 1 to Day 7. Areas I, II, and III are indicated with dashed lines. (**c**–**e**) Quantitative analysis of the relative TEWL, relative SCH and relative humidity indexes from Day 1 to Day 7 (*n* = 5). (**f**–**h**) Quantitative analysis of relative TEWL, relative SCH and relative humidity in Area II of mice RPSFs on Day 1, Day 3 and Day 7 after the surgery. (**i**) Representative HE staining of mice RPSFs from Area II on the indicated days. Scale bar: 100 μm. (**j**) Quantitative analysis of the thickness of the epidermis in Area II of mice RPSFs on Day 1, Day 3, and Day 7 after the surgery through HE staining (*n* = 3). (**k**) Representative IF staining for KLK5 (red) in Area II. Nuclei are counterstained with DAPI (blue). Scale bar: 50 μm. (**l**) Quantitative analysis of the fluorescence intensity of KLK5 in each group (*n* = 3). Data are presented as mean ± SEM. ^*^ stands for *P* < 0.05, ^**^ stands for *P* < 0.01, ^***^ stands for *P* < 0.001, ^****^ stands for *P* < 0.0001 . *RPSFs* random-pattern skin flaps, *TEWL* transepidermal water loss, *SCH* stratum corneum hydration, *KLK* kallikrein related peptidase 5

GO analysis of differentially expressed genes in mice RPSFs revealed significant enrichment in multiple pathways during skin flap necrosis, including the skin barrier pathway, water loss via skin pathway, and epidermis development pathway (Figure 1a), suggesting that RPSFs necrosis is accompanied by impairment of the skin barrier and moisture homeostasis. Therefore, skin barrier monitoring technology (Gpskin) was employed to investigate the relationship between skin barrier function and skin flap necrosis. skin barrier monitoring technology was first utilized to detect relative TEWL (RT) and relative SCH (RS) in mice RPSFs from Day 1 to Day 7 to assess its inter-observer reliability and temporal stability in skin barrier evaluation (Supplementary Figure 1b–g). The measurements obtained by Gpskin revealed no significant differences between different operators at various time points.

Notably, mice RPSFs exhibited no signs of necrosis from Day 1 to Day 3, but displayed progressively expanding necrosis from Area III to Area II from Day 4 to Day 7 (Figure 1b/Supplementary Figure 1a). No significant variations in RT, RS, or relative humidity (RH) were observed in Areas II and III from Day 1 to Day 2. However, notable divergence in these indexes emerged after Day 3 (Figure 1c–e). Separate measurements of RT, RS, and RH in Area II on Day 1, Day 3, and Day 7 further demonstrated progressive alterations (Figure 1f–h). HE and IF staining revealed significantly increased epidermal thickness accompanied by an elevated proportion of KLK5-positive keratinocytes on Day 3 and Day 7 (Figure 1i and j/Figure 1k and l).

### The mice random-pattern skin flaps necrosis was accompanied by the variations of infiltrated immune cells and activation of ferroptosis

**Figure 2:**
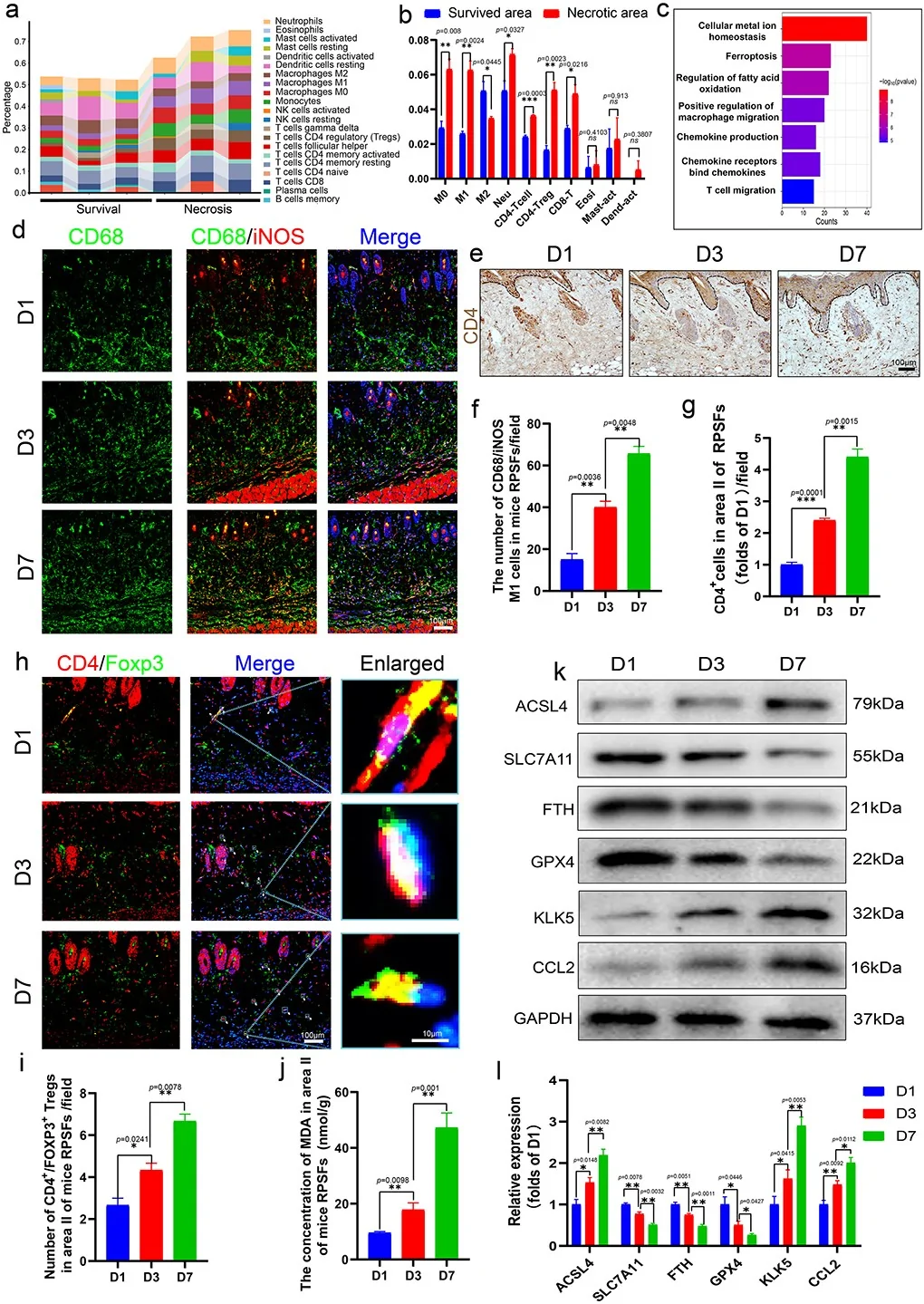
The mice RPSFs necrosis was accompanied by the variations of infiltrated immune cells and activation of ferroptosis. (**a**) The infiltration proportions of different cells according to the differentially expressed markers from the transcriptome sequencing of mice RPSFs. (**b**) Quantitative analysis of immune cells infiltration from (a) (*n* = 3). (**c**) Representative GO pathway analysis related to ferroptosis in mice RPSFs. (**d**) IF images of iNOS (red) and CD68 (green) positive M1 macrophages in Area II of mice RPSFs on Day 1, Day 3, and Day 7. Scale bar: 100 μm. (**e**) Representative IHC staining for CD4 in Area II of the mice RPSFs on Day 1, Day 3, and Day 7. Scale bar: 100 μm. (**f**) Quantification of the number of M1 macrophages in Area II of mice RPSFs in each group (*n* = 3). (**g**) Quantification of the number of CD4 positive cells in mice RPSFs in each group (*n* = 3). (**h**) Representative IF staining for CD4 (red)/Foxp3 (green) positive Tregs in Area II on Day 1, Day 3, and Day 7. Tregs were marked by white arrows and blue frame. Scale bar: 100 μm; 10 μm. (**i**) Quantification of the number of CD4/Foxp3 Tregs per field in each group (*n* = 3). (**j**) Measurement of MDA content in skin tissues from each group (*n* = 3). (**k**) Representative WB images of proteins related to ferroptosis (ACSL4, SLC7A11, GPX4, FTH), inflammation (KLK5), and chemokine (CCL2) in Area II on Day 1, Day 3, and Day 7. (**l**) The relative density values of ACSL4, SLC7A11, GPX4, FTH, KLK5 and CCL2 proteins in each group (*n* = 3). Data are presented as mean ± SEM. ^*^ stands for *P* < 0.05, ^**^ stands for *P* < 0.01, ^***^ stands for *P* < 0.001, ^****^ stands for *P* < 0.0001. *iNOS* inducible nitric oxide synthase, *ACSL4* long-chain acyl-coA synthetase 4, *FTH* ferritin heavy chain

Analysis of specific markers for different cell types in the transcriptome data of mice RPSFs revealed infiltration of various immune cells in the necrotic area of RPSFs (Figure 2a). The proportions of mononuclear macrophages, neutrophils, and T cells were significantly elevated in the necrotic area, with particularly pronounced differences in the infiltration levels of M1 macrophages and Tregs (Figure 2b). Furthermore, GO pathway analysis demonstrated significant upregulation of the ferroptosis pathway, chemokine production pathway, and chemokine receptor-ligand signaling pathway in the necrotic area (Figure 2c). Differential expression analysis of C-C motif chemokine ligands (CCLs) in mice RPSFs revealed upregulation of CCL2, CCL3, CCL17, CCL7, CCL4, and CCL22, with CCL2 exhibiting the most substantial change (Supplementary Figure 1h and i). Transcriptome analysis and qRT-PCR of C-C chemokine receptor (CCR) genes indicated that the mRNA level of CCR2 was significantly increased in necrotic RPSFs (Supplementary Figure 1j and k). Therefore, the CCL2-CCR2 axis may play a pivotal role in immune cell chemotaxis.

IF staining for CD68/iNOS revealed that the number of infiltrated M1 macrophages in Area II increased progressively from D1 to D7 (Figure 2d and f). IHC assay demonstrated a significant increase in CD4-positive cells in mice RPSFs (Figure 2e and g). Additionally, IF images further confirmed substantial migration of Tregs into Area II of skin flap (Figure 2h and i). Meanwhile, IL-35 levels gradually increased following RPSFs surgeries (Supplementary Figure 1l). Malondialdehyde (MDA) detection indicated marked elevations in lipid peroxidation during RPSFs necrosis (Figure 2j). WB analysis of Area II skin flap revealed that ferroptosis-related damage significantly increased on Days 3 and 7 post-surgery, as evidenced by downregulation of GPX4, FTH, and SLC7A11, and upregulation of ACSL4 expression, accompanied by elevated KLK5 and CCL2 levels (Figure 2k and l).

### Activation of ferroptosis impaired the skin barrier function and promoted immune cells infiltration in mice random-pattern skin flaps

**Figure 3:**
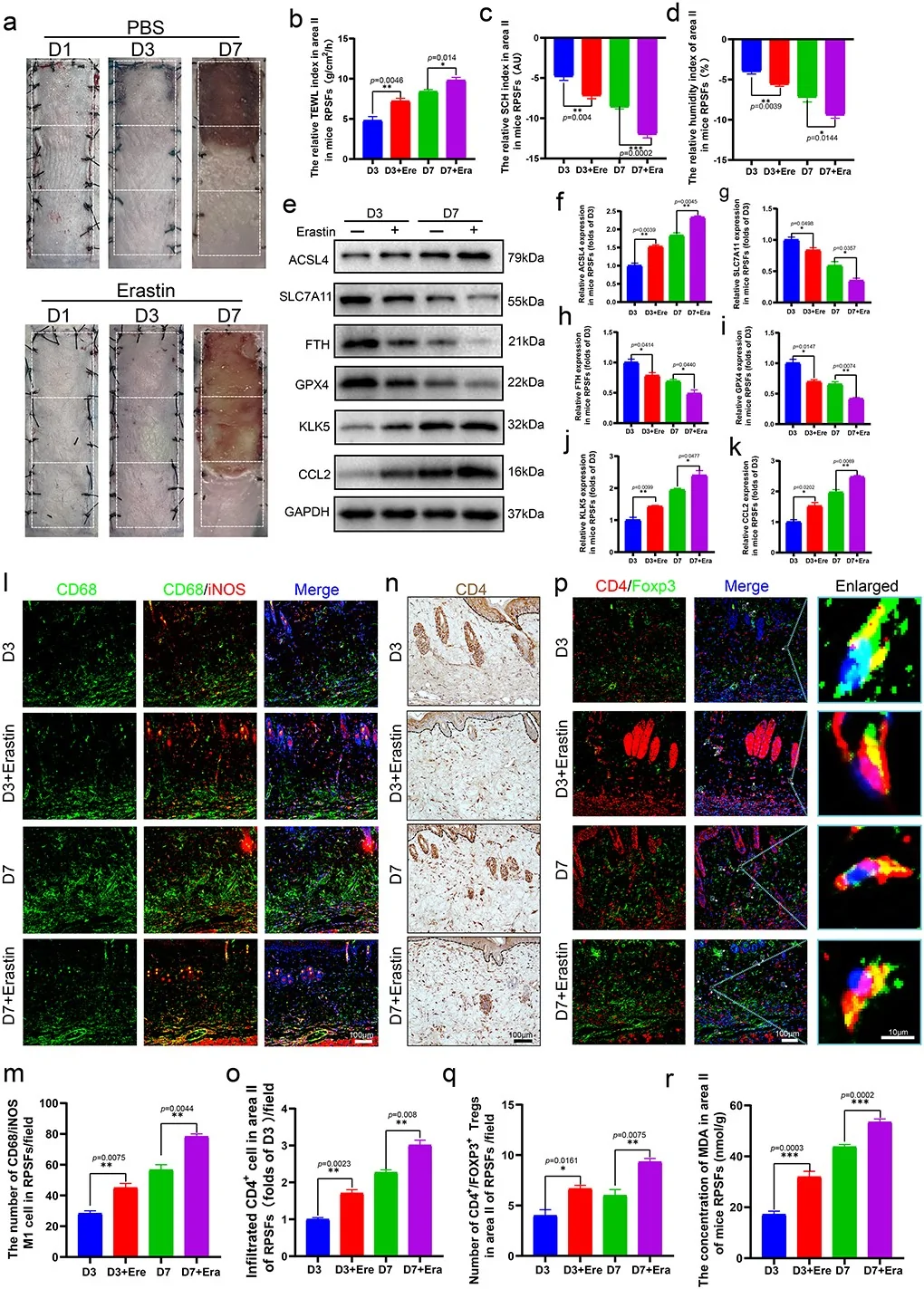
Activation of ferroptosis impaired the skin barrier function and promoted the infiltration of immune cells in mice RPSFs. (**a**) Representative photographs of RPSFs from mice treated with PBS or the ferroptosis inducer Erastin on Day 1, Day 3, and Day 7. (**b**–**d**) Quantitative analysis of relative TEWL, relative SCH and relative humidity in Area II of mice RPSFs of each group (*n* = 5). (**e**) Representative WB images of ferroptosis-related proteins (ACSL4, GPX4, FTH, SLC7A11) and inflammatory markers (KLK5), chemokine (CCL2) in Area II of D3 group, D3 + Erastin group, D7 group and D7 + Erastin group. GAPDH serves as the loading control. (**f**–**k**) The relative density values of ACSL4, SLC7A11, GPX4, FTH, KLK5, and CCL2 proteins in each group (*n* = 3). (**l**) Representative IF staining for iNOS (red) and CD68 (green) positive M1 macrophages in Area II of mice RPSFs in D3 group, D3 + Erastin group, D7 group and D7 + Erastin group (*n* = 3). Scale bar: 100 μm. (**m**) Quantification of iNOS/CD68 M1 macrophages from (i) (*n* = 3). (**n**) Representative IHC staining of CD4 positive cells in Area II of mice RPSFs in D3 group, D3 + Erastin group, D7 group and D7 + Erastin group. Scale bar: 100 μm. (**o**) Quantification of the number of CD4 positive cells in each group from (n) (*n* = 3). (**p**) Representative IF staining for CD4 (red) and Foxp3 (green) positive Tregs in Area II in D3 group, D3 + Erastin group, D7 group and D7 + Erastin group. Scale bar: 100 μm; 10 μm. (**q**) Quantification of the number of Tregs per field in each group (*n* = 3). (**r**) Measurement of MDA content in skin tissues (*n* = 3). Data are presented as mean ± SEM. PBS group stands for mice treated with PBS buffer. Erastin group stands for mice treated with Erastin (ferroptosis inducer). ^*^ stands for *P* < 0.05, ^**^ stands for *P* < 0.01, ^***^ stands for *P* < 0.001, ^****^ stands for *P* < 0.0001. *RPSFs* random-pattern skin flaps, *TEWL* transepidermal water loss, *SCH* stratum corneum hydration, *iNOS* inducible nitric oxide synthase, *ACSL4* long-chain acyl-coA synthetase 4, *FTH* ferritin heavy chain, *KLK5* kallikrein related peptidase 5, *Tregs* regulatory T cells

Since apoptosis, pyroptosis, and ferroptosis have been reported to influence RPSFs regeneration and necrosis [17–19], Ferrostatin-1 (a ferroptosis inhibitor) [20], ZVAD (an apoptosis inhibitor) [21], and Antcin A (a pyroptosis inhibitor) [22] were employed to investigate the effects of these programmed cell death pathways on the skin barrier of RPSFs. The ferroptosis inhibitor most effectively promoted the recovery of the RPSFs skin barrier (Supplementary Figure 2a–c). To further explore the relationship among ferroptosis, skin barrier function, and immune cells infiltration, Erastin (a ferroptosis inducer) was administered. On Day 1 and Day 3 post-surgery, no visible necrosis was observed in either the PBS or Erastin groups; however, apparent necrosis developed by Day 7 (Figure 3a/Supplementary Figure 2d). Measurements of RT, RS, and RH revealed that skin barrier function was impaired by Day 3, with a marked increase in TEWL on Day 7 (Figure 3b–d). WB assays confirmed that Erastin exacerbated ferroptosis and inflammation in mice RPSFs on Day 3 and Day 7, as evidenced by decreased expression of GPX4, FTH, and SLC7A11 and increased expression of ACSL4, KLK5, and CCL2 (Figure 3e–k). IF and IHC staining demonstrated that Erastin promoted M1 macrophage migration (Figure 3l and m) and infiltration of CD4-positive cells (Figure 3n and o) into skin flap on Days 3 and 7. Furthermore, the number of Tregs was also elevated (Figure 3p and q), and the concentration of IL-35 was significantly higher in Erastin-treated skin flap (Supplementary Figure 2e). MDA levels were also markedly increased in Erastin-treated skin flap (Figure 3r). Given that M1 macrophage polarization can exacerbate inflammation and necrosis, while Tregs exert anti-inflammatory and immunomodulatory effects to protect RPSFs [12], we investigated the interactions among ferroptosis, macrophages, and Tregs. We speculated that Tregs infiltration may represent a physiological negative feedback mechanism to counteract inflammation and ferroptosis in skin flap. However, this protective effect may become decompensated as ischemia intensifies.

### Exogenous Tregs inhibited the inflammation of mice random-pattern skin flaps and improved the function of skin barrier

**Figure 4:**
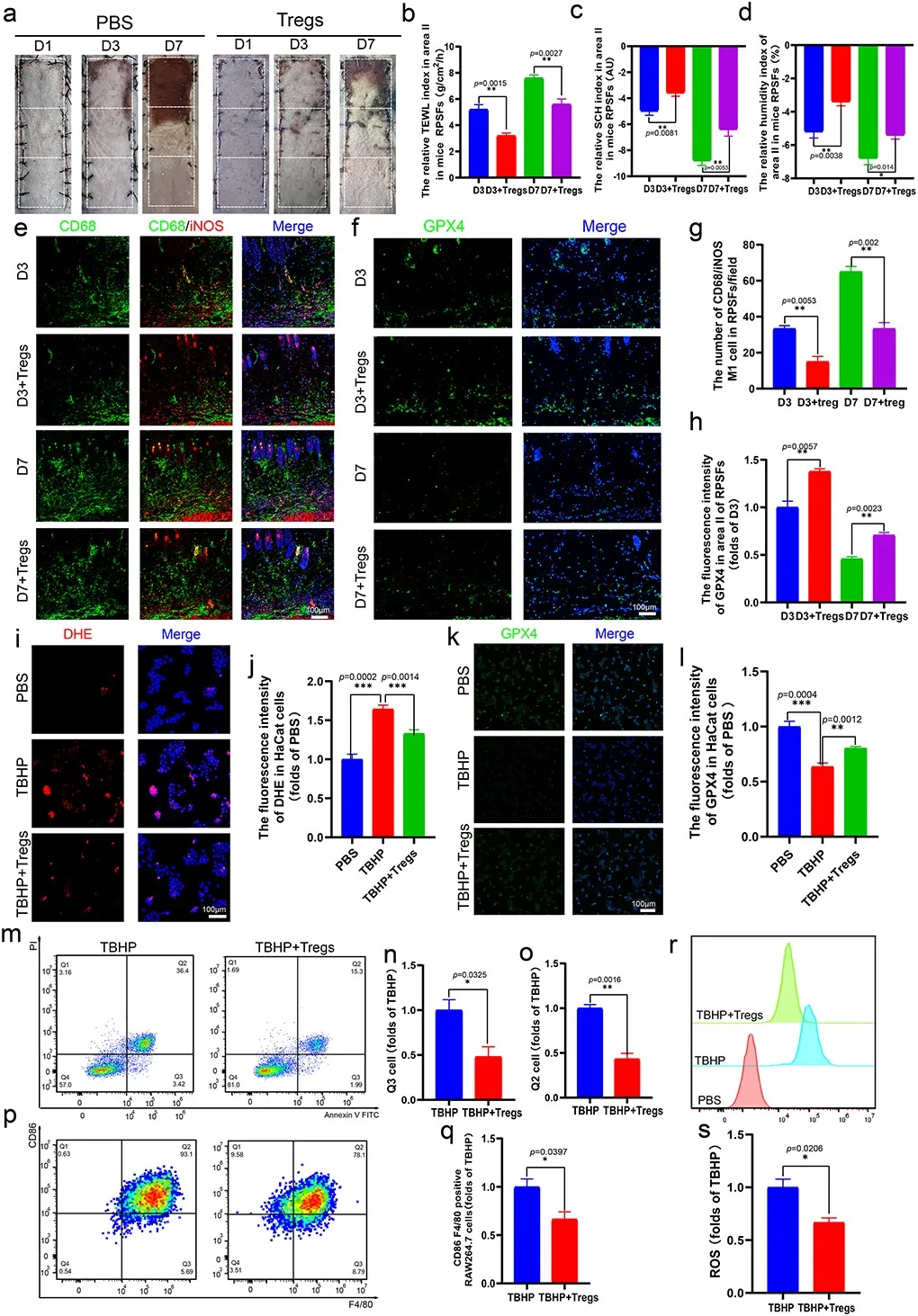
Exogenous Tregs inhibited the inflammation of mice RPSFs and improved the function of skin barrier. (**a**) Representative photographs of RPSFs from PBS-treated and Tregs-transferred mice on Day 1, Day 3 and Day 7. (**b**–**d**) Quantitative analysis of relative TEWL, relative SCH, and relative humidity indexes in Area II (*n* = 5). (**e**) Representative IF staining for iNOS (red) and CD68 (green) positive M1 macrophages in Area II after Tregs administration in D3 group, D3 + Tregs group, D7 group and D7 + Tregs group after surgery. Scale bar: 100 μm. (**f**) Representative IF staining for GPX4 (green) in Area II of D3 group, D3 + Tregs group, D7 group and D7 + Tregs group. Scale bar: 100 μm. (**g**) Quantification of the number of iNOS/CD68 M1 macrophages per field in each group (*n* = 3). (**h**) Quantification of the GPX4 fluorescence intensity in each group (*n* = 3). (**i**) Representative DHE staining (red) for reactive oxygen species (ROS) in keratinocytes. Cells were pretreated with TBHP to induce oxidative stress, followed by co-incubation with supernatant from TBHP-pretreated Tregs. Scale bar: 100 μm. (**j**) Quantification of the DHE fluorescence intensity of keratinocytes in each group (*n* = 3). (**k**) Representative IF staining for GPX4 (green) in keratinocytes under the same treatment conditions as (i). Scale bar: 100 μm. (**l**) Quantification of the GPX4 fluorescence intensity of keratinocytes in each group (*n* = 3). (**m**) FCM analysis of apoptosis in keratinocytes stained with Annexin V/PI of TBHP group and TBHP + Tregs group. (**n** and **o**) Statistical analysis of FCM of apoptotic keratinocyte (*n* = 3). (**p**) FCM analysis of M1 macrophage polarization in TBHP group and TBHP + Tregs group. (**q**) Quantification of FCM data in M1 macrophage polarization (*n* = 3). (**r** and **s**) FCM diagrams of ROS in macrophages in PBS group, TBHP group and TBHP + Tregs group (*n* = 3). Data are presented as mean ± SEM. PBS group stands for mice treated with PBS buffer. Tregs group stands for mice injected with Tregs. TBHP group stands for cells treated with TBHP (oxidative inducer). TBHP + Tregs group stands for cells treated with TBHP and Tregs supernatant. ^*^ stands for *P* < 0.05, ^**^ stands for *P* < 0.01, ^***^ stands for *P* < 0.001, ^****^ stands for *P* < 0.0001 . *F4/80* EGF-like module-containing mucin-like hormone receptor-like 1, *RPSFs* random-pattern skin flaps, *TEWL* transepidermal water loss, *SCH* stratum corneum hydration, *iNOS* inducible nitric oxide synthase, *Tregs* regulatory T cells, *ROS* reactive oxygen species

Exogenous administration of Tregs significantly diminished the necrotic area of mice RPSFs (Figure 4a/Supplementary Figure 3a) and enhanced skin barrier function, as reflected by the RT, RS, and RH indexes on Day 3 and Day 7 (Figure 4b–d). IF staining for CD68/iNOS demonstrated that Tregs markedly reduced the number of M1 macrophages on Day 3 and Day 7 (Figure 4e and g). Additionally, Tregs increased GPX4 expression in skin flap (Figure 4f and h). Tregs were pretreated with TBHP, and the conditioned medium was subsequently collected for co-culture with HaCat and RAW 264.7 cells, following previously described procedures [23]. The supernatant of TBHP-pretreated Tregs showed significantly increased concentrations of TGF-β, IL-35, and IL-10 (Supplementary Figure 3b–d). Treatment with TBHP-Tregs supernatant inhibited DHE fluorescence intensity (Figure 4i and j) and upregulated GPX4 expression (Figure 4k and l) in HaCat. FCM analysis revealed that Tregs supernatant inhibited keratinocyte apoptosis (Figure 4m–o). Furthermore, M1 polarization capacity (Figure 4p and q) and oxidative stress level (Figure 4r and s) in macrophages were attenuated by Tregs supernatant.

### The inhibition of Tregs reduced the skin barrier function of skin flaps

As the CCR2 inhibitor RS504393 has been reported to suppress T cell infiltration [24], we administered this compound to block Tregs chemotaxis. The necrotic area (Supplementary Figure 4a and b) and skin barrier function (Supplementary Figure 4c and d) of RPSFs were impaired on Day 3 and Day 7 following RS504393 treatment. WB assays revealed that RS504393 suppressed the expression of GPX4, FTH, and SLC7A11, while increasing KLK5 and ACSL4 levels (Supplementary Figure 4e–j). MDA assays confirmed enhanced lipid peroxidation following RS504393 treatment (Supplementary Figure 4k). Furthermore, the number of infiltrated Tregs (Supplementary Figure 4l and n) and GPX4 expression (Supplementary Figure 4m and o) in RPSFs were also diminished by RS504393. FOXP3-inhibiting compound (Peptide P60) [25] was employed to suppress Tregs activity. Peptide P60 is recognized as a FOXP3 inhibitor that impairs both the function and number of Tregs *in vivo* and *in vitro*. Administration of Peptide P60 significantly reduced skin flap survival and compromised skin barrier function (Supplementary Figure 4p–s). Additionally, Peptide P60 decreased the number of Tregs within skin flap (Supplementary Figure 4t and u). However, the number of M1 macrophages was unaffected by Peptide P60 treatment (Supplementary Figure 4v and w).

### M1 macrophages promoted the chemotaxis of Tregs in random-pattern skin flaps

To investigate the impact of M1 macrophages on Tregs infiltration, clodronate liposomes (CL) were injected into mice to deplete M1 macrophages. CL treatment diminished Tregs infiltration in RPSFs (Supplementary Figure 5a and b). ELISA revealed that IL-35 levels in mice skin flap was significantly decreased following CL treatment (Supplementary Figure 5c). WB assays showed that M1 macrophage depletion significantly reduced CCL2 expression in RPSFs (Supplementary Figure 5d and e), indicating that M1 macrophages partially mediate CCL2 secretion after skin flap surgeries. In conclusion, M1 macrophages promoted Tregs chemotaxis to alleviate overactive inflammation and ferroptosis in RPSFs. This phenomenon suggests that the ‘M1 macrophages-Tregs axis’ may function as a self-protective feedback mechanism during RPSFs ischemia.

### Ubiquitin specific peptidase 4 regulated the chemotaxis of Tregs to reconstruct the skin barrier of random-pattern skin flaps

**Figure 5:**
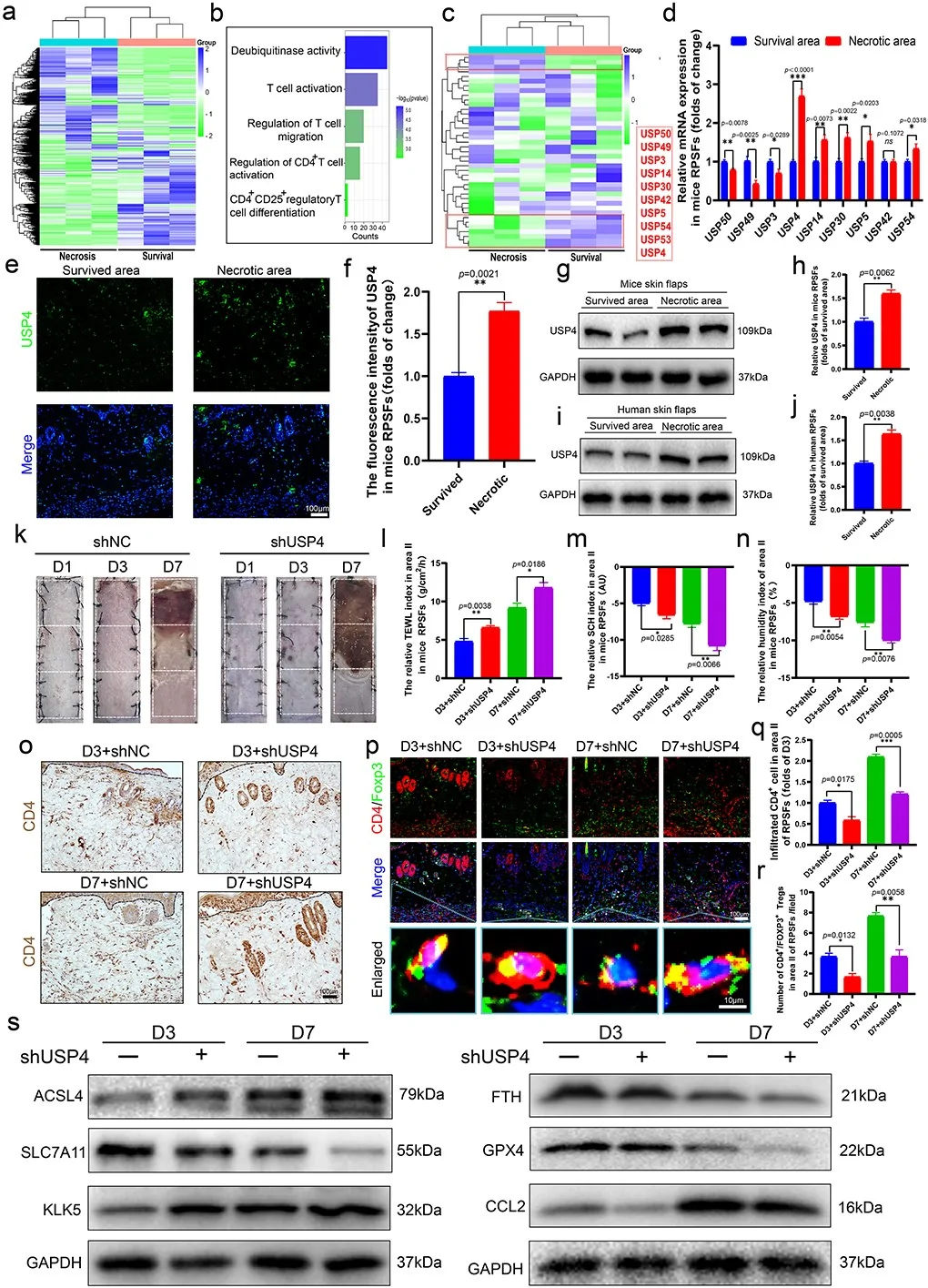
USP4 regulated the chemotaxis of Tregs to reconstruct the skin barrier of mice RPSFs. (**a**) Heatmap of differentially expressed genes from transcriptome sequencing of RPSFs. (**b**) GO pathway diagram of the differentially expressed genes in the mice RPSFs. (**c**) Heatmap of ubiquitin-specific proteases (USPs) expression from transcriptome data in the mice RPSFs. (**d**) qRT-PCR analysis of USP mRNA levels in RPSFs (*n* = 5). (**e**) Representative IF staining for USP4 (green) in RPSFs. Nuclei are stained with DAPI (blue). Scale bar: 100 μm. (**f**) Quantification of USP4 fluorescence intensity in mice RPSFs from (e) (*n* = 3). (**g**–**j**) Representative WB and quantification of USP4 in the RPSFs of mice and patients (*n* = 3). (**k**) Representative photographs of RPSFs from shNC (scrambled control) and shUSP4 (USP4 knockdown) groups on the Day 1, Day 3, and Day7. (**l**–**n**) Quantitative analysis of relative TEWL, relative SCH, and relative humidity indexes in Area II of mice RPSFs in each group (*n* = 5). (**o**) Representative IHC staining of CD4 positive cells in Area II of mice RPSFs in D3 group, D3 + shUSP4 group, D7 group and D7 + shUSP4 group. Scale bar: 100 μm. (**p**) Representative IF staining for CD4 (red) and Foxp3 (green) in Area II of mice RPSFs in D3 group, D3 + shUSP4 group, D7 group and D7 + shUSP4 group. Tregs were marked by white arrows and blue frame. Scale bar: 100 μm; 10 μm. (**q**) Quantification of the number of CD4 positive cells per field in each group (*n* = 3). (**r**) Quantification of the number of CD4/Foxp3 positive Tregs per field in each group (*n* = 3). (**s**) Representative WB analysis of key proteins in RPSFs after USP4 knockdown in mice RPSFs in each group (*n* = 3). shNC group stands for mice injected with USP4 knockdown virus control. shUSP4 group stands for mice injected with USP4 knockdown virus. Data are presented as mean ± SEM. ^*^ stands for *P* < 0.05, ^**^ stands for *P* < 0.01, ^***^ stands for *P* < 0.001, ^****^ stands for *P* < 0.0001. *RPSFs* random-pattern skin flaps, *TEWL* Transepidermal water loss, *SCH* stratum corneum hydration, *iNOS* inducible nitric oxide synthase, *ACSL4* long-chain acyl-coA synthetase 4, *KLK5* kallikrein related peptidase 5, *Tregs* regulatory T cells

GO pathway analysis revealed that deubiquitinating protease activity was significantly upregulated in necrotic RPSFs (Figure 5a and b). Gene clustering analysis was performed to identify differentially expressed USPs in skin flap (Figure 5c). qRT-PCR and IF staining verified increased expression of USP4 in the necrotic area of skin flap (Figure 5d–f). WB experiments further demonstrated that USP4 levels were elevated in the necrotic regions of both human and mouse RPSFs (Figure 5g–j). Notably, the necrotic area of mice RPSFs in the shUSP4 treatment group was significantly increased on the 7th day (Figure 5k/Supplementary Figure 6a). The RT, RS, and RH indexes indicated that skin barrier function was impaired following shUSP4 treatment (Figure 5l–n). IHC (Figure 5o and q) and IF (Figure 5p and r) assays showed that USP4 knockdown inhibited Tregs chemotaxis into Area II of skin flap. Furthermore, USP4 inhibition increased ferroptosis and inflammation levels, while decreasing CCL2 expression in RPSFs (Figure 5s/Supplementary Figure 6b–g). ELISA showed that the down-regulation of USP4 reduced the level of IL-35 in RPSFs (Supplementary Figure 6h). IF staining for CD68/iNOS confirmed that USP4 knockdown increased the number of M1 macrophages in RPSFs (Supplementary Figure 6i and j). Overexpression of USP4 in RAW 264.7 cells also suppressed TBHP-induced M1 macrophage polarization (Supplementary Figure 6k and l). CCL2 expression in RAW 264.7 cells was elevated following TBHP and Erastin treatment, whereas USP4 knockdown led to a significant reduction in CCL2, further supporting the role of USP4 in macrophage-derived CCL2 production (Supplementary Figure 6m–o).

Overall, USP4 not only directly inhibited M1 macrophage polarization but also enhanced the endogenous anti-inflammatory capacity of skin flap through the ‘M1 macrophages-Tregs axis’. Inhibition of USP4 reduced CCL2 secretion from M1 macrophages, thereby suppressing Tregs chemotaxis into the necrotic area of skin flap, which ultimately exacerbated ferroptosis and inflammatory injury.

### Ubiquitin specific peptidase 4 stabilized the TNFAIP2 protein in macrophages through deubiquitinating function

Previous studies have demonstrated that the TNF pathway modulates immune responses and inflammatory damage through regulation of cytokine and chemokine secretion [26]. To identify potential targets within the TNF pathway involved in macrophages-Tregs chemotaxis, transcriptome data were further analyzed, revealing significant differences in the expression of TNFAIP2, TNF, TNFRSF1a, TNFRSF1b, TNFRSF26, TNFRSF19, TNFRSF21, TNFRSF25, and TNFRSF18 (Supplementary Figure 5f). qRT-PCR confirmed that TNFAIP2, TNF, and TNFRSF26 were upregulated, whereas TNFRSF25 and TNFRSF18 were downregulated in necrotic RPSFs (Supplementary Figure 5g and h). WB assays confirmed upregulation of TNFAIP2 in necrotic area of mice RPSFs (Supplementary Figure 5i and j), and co-immunoprecipitation (Co-IP) proved that TNFAIP2 was a direct downstream target of USP4.

**Figure 6:**
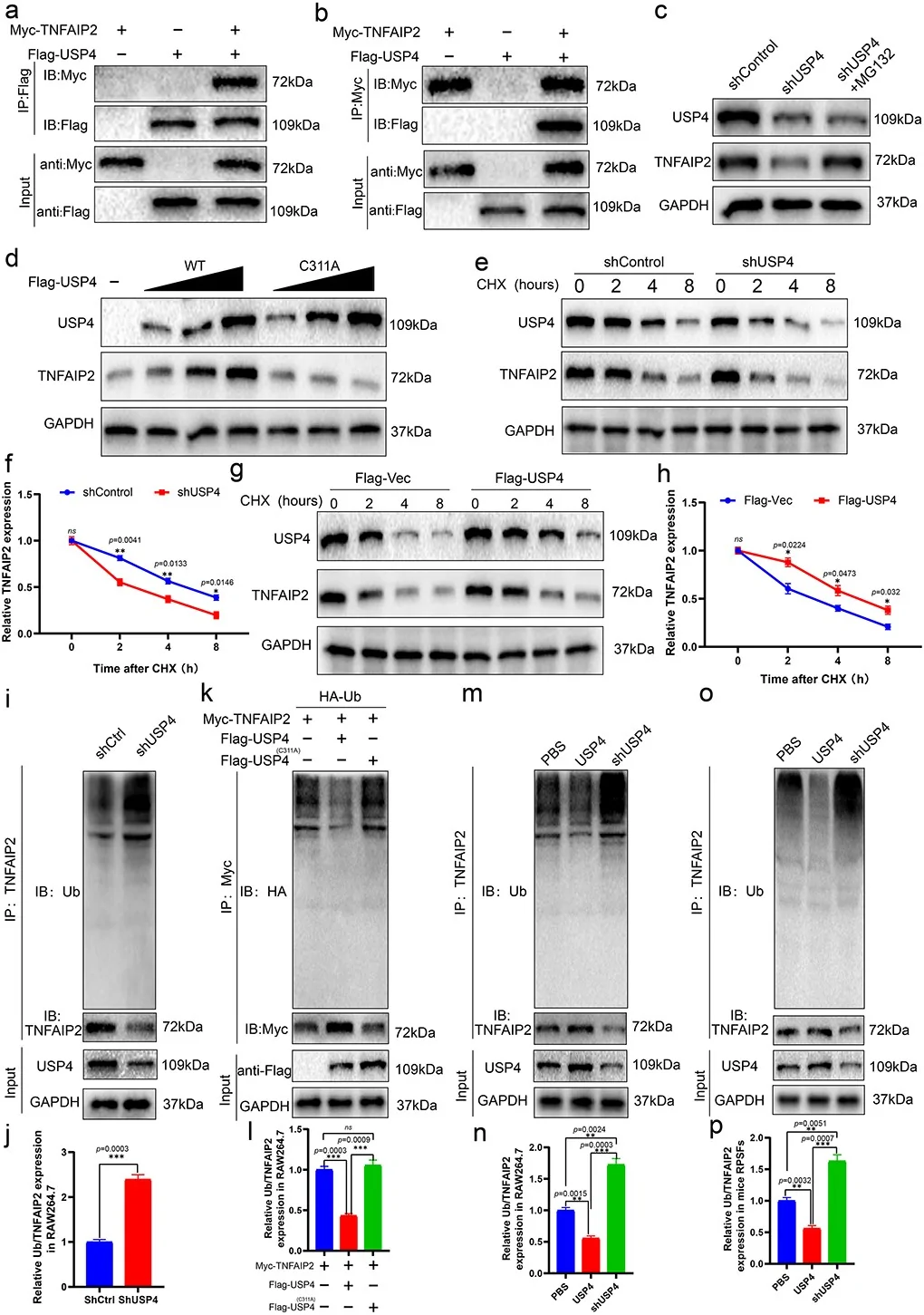
USP4 stabilized the TNFAIP2 protein in macrophages through deubiquitinating function. (**a** and **b**) Co-IP assays in RAW 264.7 macrophages. The anti-flag antibody was used for immunoprecipitation of the lysates of macrophages transfected with Myc-TNFAIP2 and flag-USP4, and the expressions of Myc-TNFAIP2 and flag-USP4 in the cells were detected. (**c**) WB analysis of USP4 and TNFAIP2 in macrophages treated with shControl, shUSP4, or shUSP4 + MG132. (**d**) WB was used to detect the expression levels of USP4 and TNFAIP2 in the WT group and C311A group. (**e**) WB analysis of TNFAIP2 and USP4 in shControl and shUSP4 macrophages treated with the protein synthesis inhibitor CHX at different time. (**f**) The relative expression levels of TNFAIP2 in macrophages of shControl and shUSP4 after CHX treatment (*n* = 3). (**g**) The expression levels of USP4 and TNFAIP2 at different time after CHX treatment in macrophages of the flag-Vec group and flag-USP4 group. (**h**) The relative expression levels of TNFAIP2 in macrophages of flag-Vec and flag-USP4 after CHX treatment (*n* = 3). (**i** and **j**) *In vitro* ubiquitination assay. IP assay was used to detect the expression levels of USP4, TNFAIP2 and Ub in the lysates of macrophages treated with shCtrl and shUSP4 (*n* = 3). (**k** and **l**) *In vitro* ubiquitination assay. IP assay was used to detect the expression levels of USP4, TNFAIP2 and Ub in the lysates of macrophages after transfection with HA-Ub, Myc-TNFAIP2, flag-USP4 and flag-USP4C311A (*n* = 3). (**m** and **n**) *In vitro* ubiquitination assay. IP assay was used to detect the expression levels of USP4, TNFAIP2 and Ub in the lysates of macrophages in PBS group, USP4 group and shUSP4 group (*n* = 3). (**o** and **p**) *In vivo* ubiquitination assay. IP assay was used to detect the expression levels of USP4, TNFAIP2, and Ub of mice RPSFs in PBS group, USP4 group, and shUSP4 group (*n* = 3). Data are presented as mean ± SEM. ^*^ stands for *P* < 0.05, ^**^ stands for *P* < 0.01, ^***^ stands for *P* < 0.001, ^****^ stands for *P* < 0.0001 and ns stands for no significant statistical difference. *TNFAIP2* tumor necrosis factor alpha induced protein 2, *IP* immunoprecipitation, *IB* immunoblotting, *USP4* ubiquitin specific peptidase 4, *RPSFs* random-pattern skin flaps

Co-IP experiments demonstrated that TNFAIP2 could be precipitated by USP4, and reverse Co-IP further confirmed that USP4 was precipitated by TNFAIP2 (Figure 6a and b). TNFAIP2 protein levels decreased following USP4 knockdown, but this reduction was reversed by treatment with the proteasome inhibitor MG132 (Figure 6c/Supplementary Figure 7b and c). To investigate whether USP4 stabilizes TNFAIP2, the catalytically inactive USP4 variant C311A was introduced into Co-IP experiments. WB analysis revealed that TNFAIP2 was stabilized by wild-type USP4, whereas its expression level was significantly reduced by the C311A mutant (Figure 6d/Supplementary Figure 7e). Although USP4 expression was reduced in skin flap and macrophages, no significant differences were observed in TNFAIP2 mRNA levels (Supplementary Figure 7d). Overexpression of USP4 inhibited TNFAIP2 degradation (Figure 6e and f), while USP4 silencing significantly accelerated TNFAIP2 degradation in the presence of the protein synthesis inhibitor cycloheximide (CHX) (Figure 6g and h).

Inhibition of USP4 increased the ubiquitination level of TNFAIP2 and decreased its intracellular protein expression (Figure 6i and j). Additionally, cells were co-transfected with HA-UB, Myc-TNFAIP2, Flag-USP4, and the mutant Flag-USP4^C311A^ to examine the regulatory effect of USP4 on TNFAIP2 ubiquitination. Overexpression of USP4 suppressed ubiquitination and stabilized TNFAIP2 levels, whereas the USP4^C311A^ mutant exerted minimal effects on TNFAIP2 ubiquitination (Figure 6k and l). Moreover, overexpression of USP4 in macrophages and RPSFs inhibited TNFAIP2 ubiquitination (Figure 6m and o), while USP4 knockdown enhanced TNFAIP2 ubiquitination (Figure 6o and p).

### Overexpression of TNFAIP2 accelerated the infiltration of Tregs and inhibited ferroptosis in mice skin flaps

**Figure 7:**
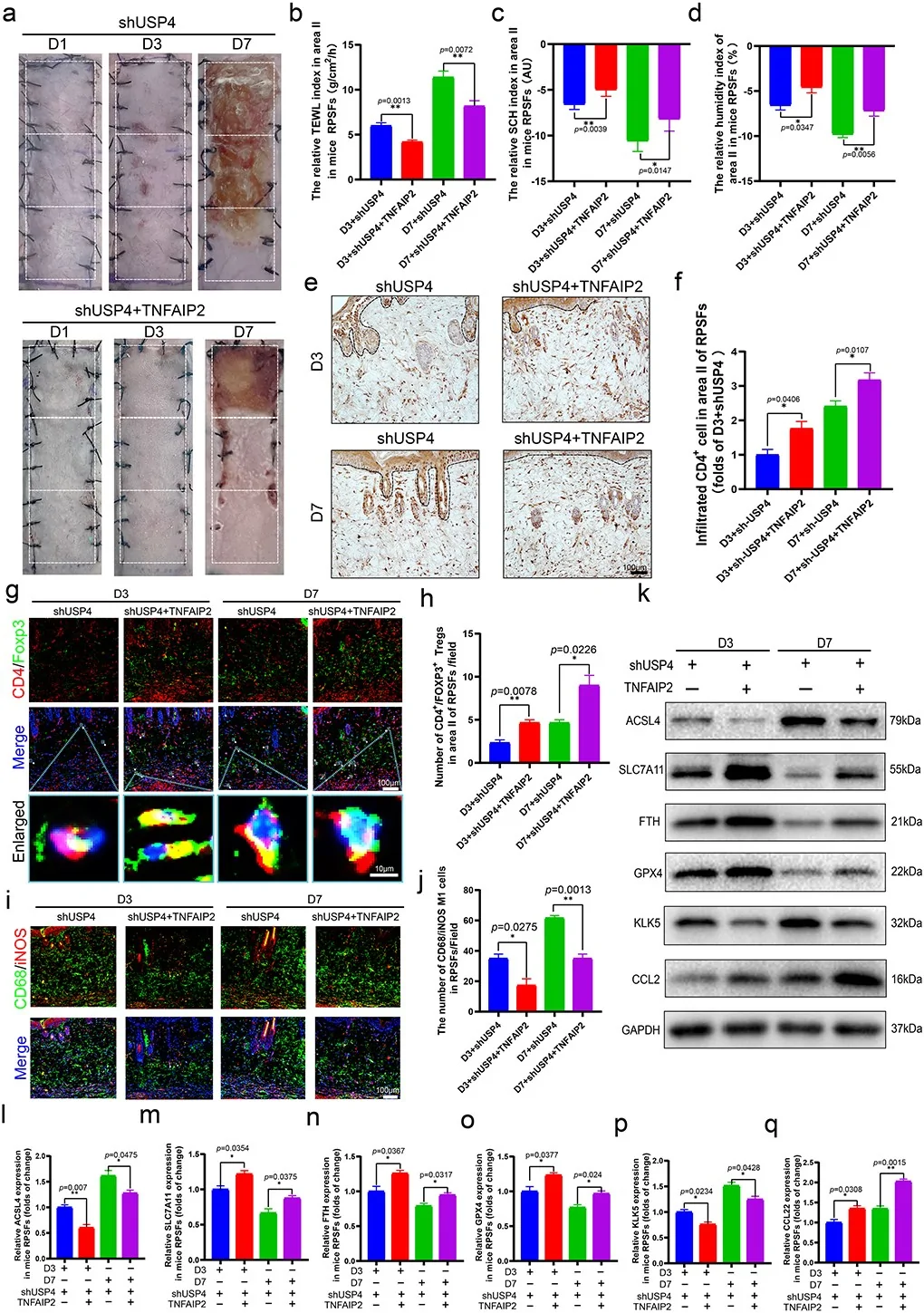
Overexpression of TNFAIP2 accelerated the infiltration of Tregs and inhibited ferroptosis in mice RPSFs. (**a**) Representative photographs of RPSFs from shUSP4 and shUSP4 + TNFAIP2 (rescue) groups on the Day 1, Day 3, and Day7. (**b**–**d**) Quantitative analysis of relative TEWL, relative SCH, and relative humidity indexes in Area II of mice RPSFs in each group (*n* = 5). (**e**) Representative IHC images of CD4 positive cells in Area II of mice RPSFs in D3 + shUSP4 group, D3 + shUSP4 + TNFAIP2 group, D7 + shUSP4 group and D7 + shUSP4 + TNFAIP2 group. (**f**) Quantitative analysis of the number of CD4 positive cells per field in each group (*n* = 3). (**g**) Representative IF staining for CD4 (red) and Foxp3 (green) in Area II in D3 + shUSP4 group, D3 + shUSP4 + TNFAIP2 group, D7 + shUSP4 group, and D7 + shUSP4 + TNFAIP2 group. (**h**) Quantitative analysis of the number of CD4/Foxp3 positive Tregs in each group (*n* = 3). (**i**) Representative IF staining for iNOS (red) and CD68 (green) in Area II in D3 + shUSP4 group, D3 + shUSP4 + TNFAIP2 group, D7 + shUSP4 group, and D7 + shUSP4 + TNFAIP2 group. (**j**) Quantitative analysis of the number of CD68/iNOS positive M1 macrophages in each group (*n* = 3). (**k**) WB assay was used to detect the expressions of ACSL4, SLC7A11, GPX4, FTH, KLK5, and CCL2 in mice RPSFs in D3 + shUSP4 group, D3 + shUSP4 + TNFAIP2 group, D7 + shUSP4 group, and D7 + shUSP4 + TNFAIP2 group. (**l**–**q**) The relative densities of ACSL4, SLC7A11, GPX4, FTH, KLK5, and CCL2 in each group (*n* = 3). Data are presented as mean ± SEM. shUSP4 group stands for mice injected with USP4 knockdown virus. shUSP4 + TNFAIP2 group stands for mice injected with USP4 knockdown virus and TNFAIP2 overexpression virus. ^*^ stands for *P* < 0.05, ^**^ stands for *P* < 0.01, ^***^ stands for *P* < 0.001, ^****^ stands for *P* < 0.0001and ns stands for no significant statistical difference.*RPSFs* random-pattern skin flaps, *TEWL* transepidermal water loss, *SCH* stratum corneum hydration, *USP4* ubiquitin specific peptidase 4, *TNFAIP2* tumor necrosis factor alpha induced protein 2, *iNOS* inducible nitric oxide synthase, *ACSL4* lLong-chain acyl-coA synthetase 4, *KLK5* kallikrein related peptidase 5, *Tregs* regulatory T cells

Overexpression of TNFAIP2 in skin flap significantly reversed the pro-necrotic effect of shUSP4 (Figure 7a/Supplementary Figure 7a). Upregulation of TNFAIP2 enhanced skin flap hydration, underscoring its protective role in maintaining skin barrier integrity (Figure 7b–d). IHC (Figure 7e and f) and IF (Figure 7g and h) assays demonstrated that TNFAIP2 facilitated Tregs infiltration. Moreover, TNFAIP2 upregulation suppressed M1 macrophage infiltration (Figure 7i and j). WB experiments confirmed that TNFAIP2 attenuated ferroptosis and inflammatory markers while enhancing CCL2 expression in RPSFs (Figure 7k–q). Furthermore, overexpression of TNFAIP2 reversed the inhibitory effect of shUSP4 on CCL2 expression in RAW 264.7. (Supplementary Figure 6m–o). To further investigate the impact of the USP4/TNFAIP2 pathway on RPSFs, we modulated the expression levels of USP4 and TNFAIP2 in mouse skin flap through targeted upregulation and downregulation. Notably, upregulation of USP4 reduced the area of skin flap necrosis and mitigated skin barrier damage, whereas downregulation of TNFAIP2 produced opposing effects (Supplementary Figure 8e–h).

### The relative transepidermal and relative stratum indexes of skin barrier might serve as clinical indicators for necrotic prediction of random-pattern skin flaps

**Figure 8:**
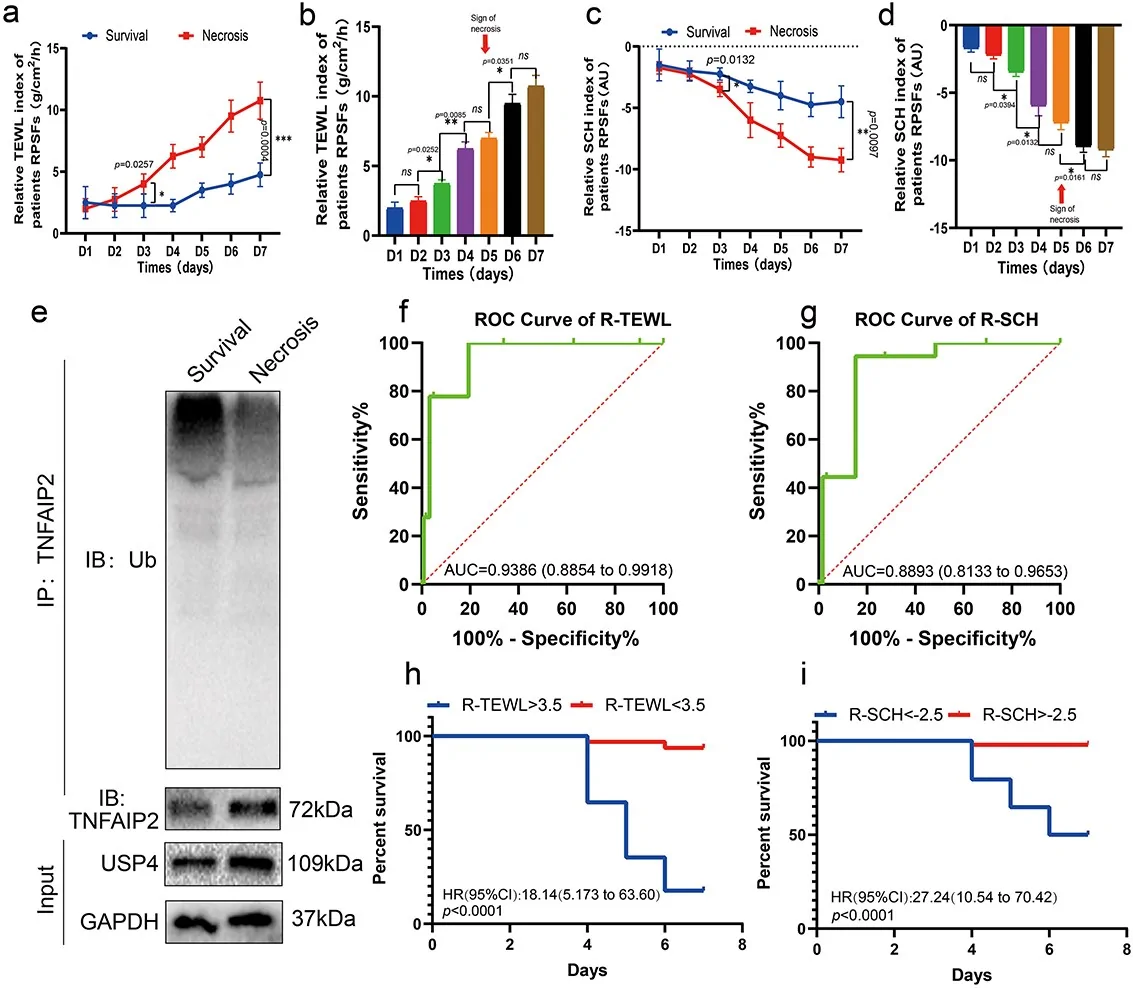
The RT and RS indexes of skin barrier might serve as clinical indicators for necrotic prediction of RPSFs. (**a** and **b**) The daily relative TEWL index of 3 representative patients who underwent RPSFs transplantation and showed necrosis on Day 5 (*n* = 3). (**c** and **d**) The daily relative SCH index of 3 representative patients who underwent RPSFs transplantation and showed necrosis on Day 5 (*n* = 3). (**e**) *In vivo* ubiquitination assay. IP assay was used to detect the expression levels of USP4, TNFAIP2 and Ub in the necrotic area and the surviving area of the patients. (**f** and **g**) ROC curves analysis of RT and RS of 80 patients who underwent RPSFs surgeries. (**h** and **i**) The survival curves of RT and RS of 80 patients who underwent RPSFs surgeries. Data are presented as mean ± SEM. ^*^ stands for *P* < 0.05, ^**^ stands for *P* < 0.01, ^***^ stands for *P* < 0.001, ^****^ stands for *P* < 0.0001 and ns stands for no significant statistical difference. *RPSFs* random-pattern skin flaps, *IP* immunoprecipitation, *IB* immunoblotting, *TEWL* transepidermal water loss, *SCH* stratum corneum hydration, *USP4* ubiquitin specific peptidase 4, *TNFAIP2* tumor necrosis factor alpha induced protein 2

It was confirmed that USP4 stabilizes TNFAIP2 to regulate the ‘M1 macrophages-Tregs axis’ in mice RPSFs by promoting CCL2 secretion, thereby facilitating spontaneous skin flap recovery and skin barrier repair. In the early stages of mice RPSFs, no visible signs of necrosis were present, yet skin barrier function was already impaired. Therefore, we hypothesized that alterations in skin barrier indexes mediated by the USP4/TNFAIP2 pathway could serve as predictive markers for clinical skin flap necrosis. Three patients who developed ischemic necrosis following RPSFs surgeries were enrolled. Although no macroscopic necrosis was detectable on Day 3, significant alterations in RT and RS indexes were observed at this early stage (Figure 8a–d). Furthermore, WB analysis of patients’ RPSFs samples revealed patterns similar to those observed in animal experiments, with increased USP4 expression and reduced TNFAIP2 ubiquitination in necrotic areas (Figure 8e/Supplementary Figure 7f).

Subsequently, the patient sample size was expanded. A total of 80 patients were included, of whom 18 developed RPSFs necrosis (Supplementary data, Table 1). Receiver operating characteristic (ROC) curves analysis identified the optimal threshold values was 3.5 for RT [area under the curve (AUC) = 0.9386, 95% CI 0.8854 to 0.9918] and −2.5 for RS (AUC = 0.8893, 95% CI 0.8133 to 0.9653) in predicting skin flap necrosis (Figure 8f and g). Survival curves analysis revealed that skin flap with RT >3.5 and RS < −2.5 were significantly more likely to undergo subsequent ischemic necrosis (Figure 8h and i).

Furthermore, the patients who developed RPSFs necrosis typically exhibited such index changes within the first three or four days after surgery, and the absolute values of RT and RS gradually increased over the following days. Ischemia occurs immediately after skin flap surgeries, while 72 hours post-surgery represents a critical time point for the regeneration of occluded blood vessels [27]. Thus, based on our clinical experiments, we speculated that the first three days after surgery might constitute a window period for assessing the future outcome of RPSFs using skin barrier monitoring technology.

A prospective clinical study was conducted to validate the clinical utility of RT and RS on Day 3 after RPSFs surgeries and to investigate the benefits of this technology. Patients (Supplementary data, Table 2) of the two groups (Group A and Group B) who underwent RPSFs surgeries exhibited significant changes in RT and RS indexes (Supplementary data, Tables 3.1 and 3.2). Notably, while no significant differences were observed in the number of patients developing skin flap necrosis between the two groups (χ^2^ = 0.2163, *P* = 0.6418) (Supplementary data, Table 4), the final survival area of the RPSFs that developed necrosis was significantly higher in Group A compared to Group B (Supplementary Figure 10a). Moreover, it was observed that skin barrier indexes of patients who developed RPSFs necrosis showed significant differences on Day 3 in both groups. Furthermore, the changes in these indexes became more pronounced over time in Group B than in Group A (Supplementary Figure 10b and c). A possible explanation is that skin barrier monitoring technology-guided assessment enabled earlier prediction of RPSFs necrosis, facilitating advanced clinical intervention to reduce necrotic area and skin barrier injury. Furthermore, according to the data of Group B, the sensitivity and specificity of RT (>3.5) for predicting RPSFs necrosis were 91.67% (64.06%–98.52%) and 90.91% (76.93%–96.94%) (Supplementary data, Table 5.1). And the sensitivity and specificity of RS (<−2.5) for predicting RPSFs necrosis were 83.33% (55.20%–97.06%) and 87.88% (72.91%–95.78%) (Supplementary data, Table 5.2). These results demonstrated that both RT and RS exhibited favorable sensitivity and specificity in clinical diagnosis. In summary, the application of skin barrier monitoring technology may assist clinicians in early prediction of potentially necrotic RPSFs, enabling timely intervention and thereby improving skin flap survival rates.

## Discussion

The blood supply of RPSFs primarily depends on diffusely distributed subcutaneous vessels, which inherently leads to unstable perfusion [28]. Necrosis in RPSFs typically occurs following a delayed phase of ischemia, presenting considerable challenges for early prognosis. Current clinical assessments of skin flap viability mainly rely on subjective observations of color, texture, and edema, which often result in inaccurate judgments. Invasive methods, including puncture, tissue biopsy, or angiography, are also employed. However, these approaches are frequently associated with excessive injury. Therefore, developing a noninvasive strategy to predict potential necrosis in RPSFs is urgently needed.

Ischemia, hypoxia, and ferroptosis inhibit keratinocyte activity and compromise skin barrier function [29–31], leading us to hypothesize that skin barrier indexes could serve as early indicators of skin flap necrosis. Studies have demonstrated that the skin barrier monitoring technology (Gpskin) exhibits high reliability in assessing skin barrier function. This device not only displays a strong positive correlation with measurements from standardized skin barrier devices such as Aquaflux, Epsilon, Biox AquaFlux, and Courage-Khazaka Corneometer but also demonstrates inter-observer reliability, as evidenced by the absence of statistically significant differences between measurements from different observers [32–34]. The device represents a flexible and cost-effective option that offers greater ease of use compared to other available instruments. These advantages enhance its clinical utility and potential for widespread adoption. Furthermore, the inter-observer reliability and temporal stability of Gpskin were also validated in a mice RPSFs model, leading us to select this device for skin barrier measurement.

Keratinocytes play a pivotal role in maintaining skin barrier functions [35], and overexpression of KLK5 in keratinocytes exacerbates skin barrier damage through activation of inflammatory signaling [36]. Additionally, excessive keratinocyte death-induced skin barrier damage manifests as epidermal thickening in cutaneous inflammation [37]. The observed epidermal thickening and elevated KLK5 expression in the RPSFs model were consistent with previous discoveries, indicating that keratinocyte injury and barrier disruption may be interrelated. Cutaneous moisture levels serve as a primary indicator of skin barrier function [38]. Therefore, the relative indexes of TEWL, SCH, and humidity were employed to assess skin barrier function in mouse RPSFs. Significant aberrations in RT, RS, and RH values were detected prior to macroscopic RPSFs necrosis, and GO pathway analysis further corroborated that the process of RPSFs necrosis is accompanied by alterations in skin moisture and barrier function. Consequently, monitoring these indexes provides a strategy for predicting the underlying biological state of RPSFs.

Ferroptosis represents a form of iron-dependent programmed cell death characterized by intracellular iron accumulation and lipid peroxidation [39]. Emerging evidence has indicated that activation of ferroptosis impairs keratinocyte function and promotes M1 macrophage polarization [40, 41]. The mechanistic investigation in the present study also revealed that ferroptosis emerged as the predominant mode of programmed cell death affecting the skin barrier. This finding is particularly significant during the process of inflammatory responses in RPSFs. Furthermore, the crosstalk between ferroptosis and inflammation established a self-amplifying loop wherein Erastin not only directly induced ferroptotic damage in keratinocytes but also promoted M1 macrophage polarization, which in turn amplified inflammation and tissue injury. This observation explains the potent protective effect of Ferrostatin-1 in inhibiting ferroptosis as a key therapeutic axis. Thus, attenuating the inflammatory burden of M1 macrophages and suppressing ferroptosis may represent promising therapeutic strategies for RPSFs recovery.

Tregs can promote keratinocyte recovery, thereby accelerating skin barrier restoration [42, 43]. Evidence has demonstrated that IL-35 secreted by Tregs inhibits M1 macrophage polarization to exert anti-inflammatory effects [44, 45]. Additionally, CCL2 serves as a key chemokine for Tregs, facilitating their infiltration into injured sites via CCR2 engagement [46]. Exogenous administration of Tregs has been shown to promote skin barrier recovery and reduce the abundance of polarized M1 macrophages in RPSFs, whereas inhibition of Tregs chemotaxis and function using RS504393 and Peptide P60 aggravated skin barrier injury. Consistent with previous reports [11], specific reduction in Tregs numbers impaired skin barrier recovery capacity. Moreover, depletion of M1 macrophages also led to diminished Tregs infiltration in skin flap, collectively supporting the concept of an ‘M1 macrophages-Tregs axis’ as a self-protective feedback mechanism in RPSFs necrosis. Mechanistically, ischemia initially induces M1 macrophage polarization and ferroptosis, while simultaneously triggering a compensatory response wherein M1 macrophages facilitate Tregs recruitment via CCL2 secretion. These recruited Tregs subsequently counteract tissue damage by suppressing M1 macrophage activity and directly promoting keratinocyte recovery. However, the lack of specificity of the chemical inhibitors (RS504393 and Peptide P60) employed to target Tregs chemotaxis and function represents a limitation that should be acknowledged. Despite this constraint, the consistent trends between our inhibitor-based data and prior findings from Tregs-specific depletion studies [11] partially support our conclusion that Tregs play a pivotal role in regulating the ‘M1 macrophages-Tregs axis’ and skin barrier repair in RPSFs. These results provide preliminary but meaningful evidence for the functional relevance of Tregs in skin flap models.

USP4 was identified as a key upstream regulator of the M1 macrophages-Tregs axis, establishing a molecular link between ischemic stress and immune modulation. Evidence has demonstrated that USP4 promotes fibroblast proliferation and migration during cutaneous regeneration [47]. Although USP4 enhanced the immunosuppressive function of Tregs by regulating interferon regulatory factor 8 (IRF8) [48], its roles in skin barrier maintenance and Tregs migratory behavior remained poorly elucidated. The present findings have demonstrated that USP4 upregulation may represent a key adaptive response. Upregulated USP4 intrinsically inhibits M1 polarization of macrophages while extrinsically enhancing CCL2 production to facilitate Tregs recruitment through stabilization of TNFAIP2.

Transcriptome sequencing and Co-IP analysis identified TNFAIP2 as a direct downstream target of USP4. TNFAIP2 serves as an important component in the activation of inflammatory and oxidative pathways [49–51]. Furthermore, studies have indicated that TNFAIP2 promotes transendothelial migration of T cells [52]. However, the function of TNFAIP2 in RPSFs regeneration, skin barrier maintenance, and Tregs migration remained undefined. The present study suggests that TNFAIP2, stabilized by USP4 within M1 macrophages, exerts a protective function by driving the pro-resolutive ‘M1 macrophages-Tregs axis’ through CCL2 secretion in RPSFs, indicating that TNFAIP2 counteracts inflammation and necrotic damage through regulation of multiple cell types. This mechanism broadens the understanding of TNFAIP2 functions in transcellular anti-inflammatory effects.

In conclusion, the ‘M1 macrophages-Tregs axis’ in mice RPSFs is regulated by the USP4/TNFAIP2 pathway. Ischemia activates M1 macrophage polarization and ferroptosis following RPSFs surgeries. Concurrently, a self-responsive upregulation of USP4 serves as a compensatory mechanism that suppresses pro-inflammatory M1 macrophage polarization and enhances CCL2 expression to promote Tregs infiltration through stabilization of TNFAIP2 within M1 macrophages.

The relevance between basic research and clinical translation is underscored in this study. Most importantly, the clinical investigation validated that skin barrier dysfunction, which occurs as an early event prior to the appearance of skin flap necrosis, can be predicted through skin barrier monitoring. The thresholds for RT and RS derived from ROC and survival curves provide objective, quantifiable criteria for risk stratification. The intervention trial further suggested that clinical outcomes were significantly improved upon early warnings provided by the skin barrier monitoring technology. This proposed strategy may enhance both the timeliness and accuracy of diagnostic decision-making by surgeons.

The present study established a non-invasive clinical tool for predicting RPSFs necrosis through skin barrier detection, offering a direct solution for early postoperative assessment. This methodology holds potential to assist clinicians in rapidly identifying RPSFs susceptible to necrosis, ultimately improving clinical management efficiency in future healthcare settings. Under the guidance of skin barrier monitoring technology, clinicians could identify potential risk of RPSFs necrosis at an earlier stage, enabling timely clinical interventions to promote survival rates and reduce additional patient harm. Furthermore, our findings elucidated the regulatory role of the USP4/TNFAIP2 axis in maintaining M1 macrophage and Tregs homeostasis, providing a theoretical foundation for future precision medicine approaches in ischemic skin flap.

In addition to RPSFs, pedicled flap and perforator flap are also commonly employed for reconstruction of various skin defects in clinical practice. However, only RPSFs surgeries were included in the present study. Consequently, future investigations will encompass a broader range of skin flap types to examine the association between skin barrier function and potential necrosis. Clinical patients undergoing skin flap surgeries frequently present with underlying comorbidities such as diabetes mellitus, hypertension, or atherosclerosis, which may significantly affect angiogenesis and immune responses within skin flap. Future studies employing disease-specific animal models would strengthen the translational potential and external validity of these findings.

Although the chemical inhibitors employed (RS504393 and Peptide P60) effectively modulated Tregs-related phenotypes and yielded results consistent with expected biological outcomes, their potential non-specificity cannot be excluded. To address this limitation and validate our findings more rigorously, future studies will incorporate genetically modified mouse models. Such genetic approaches will eliminate off-target effects associated with chemical inhibitors, enabling more definitive confirmation of the ‘M1 macrophages-Tregs axis’ and enhancing the translational potential of our discoveries.

## Conclusions

This study elucidated the role of the USP4/TNFAIP2 pathway in regulating the ‘M1 macrophages-Tregs axis’ to influence skin barrier function, thereby addressing a critical gap in understanding the interactive mechanisms between M1 macrophages and Tregs in RPSFs. Building upon this mechanistic insight, we proposed a novel concept that skin barrier assessment could serve as a predictive indicator for RPSFs necrosis, offering a feasible approach for intelligent postoperative management following skin flap surgeries. Clinical experiments further demonstrated a correlation between quantified skin barrier function and RPSFs necrosis, establishing a new diagnostic modality for clinical prediction of skin flap necrosis. Nevertheless, this experimental work primarily focused on RPSFs, with other types of skin flaps not included in the investigation. Therefore, future research will be expanded to explore the relationship between skin barrier function and additional skin flaps types.

## Supplementary Material

Supplementary_material_tkag016

## Data Availability

The data that support the findings of this study are available from the corresponding author upon reasonable request. Some data may not be made available because of privacy or ethical restrictions.
