# Supplementary material for: Innovative research on predicting skin flaps necrosis through quantification of the USP4/TNFAIP2 regulated ‘M1 macrophages-Tregs axis’ via skin barrier monitoring technology
Source: Burns Trauma. 2026 Feb 27;14:tkag016. doi: 10.1093/burnst/tkag016 (PMC13602267; doi:10.1093/burnst/tkag016)
Supplement: Supplementary file 1 [file supplementary_material_tkag016.zip › supplementary Table Antibodies list.docx]

**Antibodies list for western blotting**

| Name | Concentration | **Catalog Number** | Origin |
| --- | --- | --- | --- |
| Anti-ACSL4 | 1:1000 | 22401-1-AP | Proteintech |
| Anti-SLC7A11 | 1:1000 | 26864-1-AP | Proteintech |
| Anti-GPX4 | 1:1000 | 67763-1-Ig | Proteintech |
| Anti-FTH | 1:1000 | 381204 | Zenbio |
| Anti-KLK5 | 1:1000 | A2991 | Abclonal |
| Anti-CCL2 | 1:1000 | A23288 | Abclonal |
| Anti-USP4 | 1:1000 | 66822-1-Ig | Proteintech |
| Anti-TNFAIP2 | 1:1000 | sc-28318 | Santa Cruz |
| HRP-conjugated Goat Anti-Rabbit IgG | 1:2000 | SA00001-2 | Proteintech |
| HRP-conjugated Goat Anti-Mouse IgG | 1:2000 | SA00001-1 | Proteintech |

**Antibodies list for immunofluorescence and immunohistochemistry**

| Name | Concentration | **Catalog Number** | Origin |
| --- | --- | --- | --- |
| anti-KLK5 | 1:200 | A2991 | Abclonal |
| anti-CD4 | 1:200 | 250015 | Zenbio |
| anti-Foxp3 | 1:200 | 65089-1-Ig | Proteintech |
| anti-CD68 | 1:200 | 28058-1-AP | Proteintech |
| anti-iNOS | 1:200 | 22226-1-AP | proteintech |
| anti-GPX4 | 1:200 | 67763-1-Ig | proteintech |
| anti-USP4 | 1:200 | 66822-1-Ig | proteintech |
