# Supplementary material for: Innovative research on predicting skin flaps necrosis through quantification of the USP4/TNFAIP2 regulated ‘M1 macrophages-Tregs axis’ via skin barrier monitoring technology
Source: Burns Trauma. 2026 Feb 27;14:tkag016. doi: 10.1093/burnst/tkag016 (PMC13602267; doi:10.1093/burnst/tkag016)
Supplement: Supplementary file 1 [file supplementary_material_tkag016.zip › supplementary Table PCR primers list.docx]

**PCR primers list**

| Name | Sequence |
| --- | --- |
| USP49-F | 5′-CATCCCCTTCTCCCAGAGGA-3′ |
| USP49-R | 5′-ATGTGA CCTGACTGAGCAGC-3′ |
| USP4-F | 5′-TACTAAACTGGTACGGCTGTGT-3′ |
| USP4-R | 5′-GGGATGTTGAATAGCTTCCGC-3′ |
| USP3-F | 5′-AGCAAGATAAAGTTCAGCACACA-3′ |
| USP3-R | 5′-ACTTTCTGTACCAGTCCCAGC-3′ |
| USP14-F | 5′-GTTGGAGCTTGGCTGAAGAC-3′ |
| USP14-R | 5′- CTGCATTCAGCATCC AAAGA-3′ |
| USP30-F | 5′-AGCAATAGCTGACGGACCAC-3′ |
| USP30-R | 5′-TG AAAACCAAGCAGCCCCA-3′ |
| USP5-F | 5′-CCACGAACAATAGTTTAGAACG-3′ |
| USP5-R | 5′-AGGTCCCACTGGCACAGA-3′ |
| USP54-F | 5′-GAGTTAGAGGCAGCGAAAGGGT-3′ |
| USP54-R | 5′-TGGGAAAGCGACGGAGATTGAG-3′ |
| USP50-F | 5′-GACATCCAGGGCACAGTAAAGAG-3′ |
| USP50-R | 5′-GACCACCATCCAGATCTCCAAAG-3′ |
| USP42-F | 5′-CAGAGTGTTACCAAGGCGTTAGA-3′ |
| USP42-R | 5′-GCGCTTCAGTGAGATGGTAAGAA-3′ |
| CCL2-F | 5′-TCAGCCAGATGCAATCAATGCC-3′ |
| CCL2-R | 5′-GCTTCTTTGGGACACTTGCT-3′ |
| CCL3-F | 5′-CATATGGAGCTGACACCCCG-3′ |
| CCL3-R | 5′-GAGCAAAGGCTGCTGGTTTC-3′ |
| CCL7-F | 5′-CTCGACCCACTTCTGATGGG-3′ |
| CCL7-R | 5’-CCCTGGGAAGCTGTTATCTTCAA-3′ |
| CCL4-F | 5′-GCTT CCTCGCAACTTTGTGG-3′ |
| CCL4-R | 5′-TCACTGGGATCAGCACAGAC-3′ |
| CCL17-F | 5′-CGAGAGTGCTGCCTGGATTACT-3′ |
| CCL17-R | 5′-GGTCTGCACAGATGAGCTTG CC-3′ |
| CCL22-F | 5′-GTGGAAGACAGTATCTGCTGCC-3′ |
| CCL22-R | 5′-AGGCTTGCGGCAGGATTTTGAG-3′ |
| CCR2-F | 5′-ACTTGCAT GGCATTTACAGGT-3′ |
| CCR2-R | 5′-ACTCCTGGCTGGAAAAGAAGA-3′ |
| CCR7-F | 5′-ATTTGTTTCGTGGGCCTACTG-3′ |
| CCR7-R | 5′- TCATGGTCTTGAG CCTCTTGA-3′ |
| CCRL2-F | 5′-CCTGGTTGTGCTTATCCTGGT-3′ |
| CCRL2 | 5′-AGAATTTTACACATGGGATCGCC-3′ |
| CCR5-F | 5′-ATCTGGCA TAGTCT CATCTGGC-3′ |
| CCR5-R | 5′-GGCTGCGATTTGCTTCACAT-3′ |
| TNF-F | 5′-CAGGCGGTGCCTATGTCTC-3′ |
| TNF-R | 5′-GCTACGACGTGGGCTACAG-3′ |

**PCR primers list**

| Name | Sequence |
| --- | --- |
| TNFRSF1a | 5'-GTGCTGTTGCCTCTGGTTATCT-3′ |
| TNFRSF1a | 5'-GAGACAGGATGACTGAAGCGTG-3' |
| TNFRSF1b | 5′-CGGGCCAACA T GCAAA AGTC-3′ |
| TNFRSF1b | 5′-CAGATGCGGTTCTGTTCCC-3′ |
| TNFRSF21 | 5′-ATTGGCACATACCGCCATGTT-3′ |
| TNFRSF21 | 5′-GGCTTGTGTTGGTACAATGCTC-3′ |
| TNFRSF19 | 5′-GACCTCAGCTCCACGAATATG-3′ |
| TNFRSF19 | 5′-CACCCCACAACCAAGAGTCG-3′ |
| TNFRSF18 | 5′-ACCCAGTTCGGGTTTCTCAC-3′ |
| TNFRSF18 | 5′-CCAGATGTGCAGTCCAAGC-3′ |
| TNFAIP2 | 5'-CCTGCTCTCCCTACGC-3′ |
| TNFAIP2 | 5'-CGTCCAAGATGCTCCG-3′ |
| TNFRSF26 | 5′-CAGAGTCGTCAGGAAATGCAATG-3′ |
| TNFRSF26 | 5'-AGCGCCAATGATGATGATGAAAG-3′ |
| TNFRSF25 | 5'-GGCTACAACACCAGTCCATGAG-3′ |
| TNFRSF25 | 5'-TACAAGGCTGCCATCGACAATAT-3′ |
| GAPDH | 5'-GGGAGCCAAAAGGGTCATCATCTC-3' |
| GAPDH | 5'-CCATGCCAGTGAGCTTCCCGTTC-3' |
