# Supplementary material for: Innovative research on predicting skin flaps necrosis through quantification of the USP4/TNFAIP2 regulated ‘M1 macrophages-Tregs axis’ via skin barrier monitoring technology
Source: Burns Trauma. 2026 Feb 27;14:tkag016. doi: 10.1093/burnst/tkag016 (PMC13602267; doi:10.1093/burnst/tkag016)
Supplement: Supplementary file 1 [file supplementary_material_tkag016.zip › supplementary Table2.docx]

**Table2.**

****Patients’ characteristics****

|  | Group A (n=45) | Group B (n=45) |
| --- | --- | --- |
| Age (y) | 43.09 ± 11.47 | 46.73 ± 9.62 |
| Gender |  |  |
| Male (%) | 23 (51.11) | 23 (51.11) |
| Female (%) | 22 (48.89) | 22 (48.89) |
|  |  |  |
| Situ of RPSFs |  |  |
| Neck (%) | 4 (8.89%) | 3 (6.67%) |
| **Upper limb** (%) | **10 (22.22%)** | **11 (24.44%)** |
| **Lower limb** (%) | **18 (40%)** | **16 (35.56%)** |
| **Abdomen** (%) | **4 (**8.89%**)** | **6 (13.33%)** |
| **Back** (%) | **5 (11.11%)** | **4 (**8.89%**)** |
| **Chest** (%) | **4 (**8.89%**)** | **5 (11.11%)** |
