# Supplementary material for: Innovative research on predicting skin flaps necrosis through quantification of the USP4/TNFAIP2 regulated ‘M1 macrophages-Tregs axis’ via skin barrier monitoring technology
Source: Burns Trauma. 2026 Feb 27;14:tkag016. doi: 10.1093/burnst/tkag016 (PMC13602267; doi:10.1093/burnst/tkag016)
Supplement: Supplementary file 1 [file supplementary_material_tkag016.zip › supplementary Table3.1.docx]

**Table3.1**

| No. | D1 | | D2 | | D3 | | D4 | | D5 | | D6 | | D7 | |  |  |
| --- | --- | --- | --- | --- | --- | --- | --- | --- | --- | --- | --- | --- | --- | --- | --- | --- |
|  | RT | RS | RT | RS | RT | RS | RT | RS | RT | RS | RT | RS | RT | RS | Outcome | SR (%) |
| 1 | 1, 0 | | 1, -1 | | 1, 0 | | 2, -1 | | 2, -1 | | 3, -3 | | 3, -2 | | Survival | 92.48 |
| 2 | 0, 0 | | 1, -1 | | 2, -2 | | 1, -1 | | 2, -2 | | 2, -3 | | 2, -2 | | Survival | 92.57 |
| 3 | 2, -1 | | 3, -2 | | 3, -1 | | 2, -2 | | 1, -3 | | 2, -2 | | 2, -3 | | Survival | 95.36 |
| 4 | 1, -2 | | 2, -1 | | 0, -2 | | 2, -2 | | 3, -2 | | 2, -3 | | 4, -2 | | Survival | 92.05 |
| 5 | 1, 0 | | 2, 0 | | 2, -2 | | 3, -1 | | 2, -3 | | 2, -2 | | 3, -3 | | Survival | 95.62 |
| 6 | 2, -1 | | 2, -1 | | 3, -1 | | 2, -1 | | 3, -2 | | 3, -3 | | 4, -2 | | Survival | 92.51 |
| 7 | 3, -2 | | 3, -2 | | 4, -2 | | 4, -3 | | 3, -3 | | 3, -2 | | 3, -4 | | Survival | 93.90 |
| 8 | 2, -1 | | 1, -3 | | 2, -1 | | 3, -2 | | 2, -2 | | 3, -2 | | 4, -2 | | Survival | 92.47 |
| 9 | 1, -2 | | 2, -2 | | 2, -2 | | 3, -3 | | 2, -1 | | 3, -2 | | 4, -3 | | Survival | 93.61 |
| 10 | 0, 0 | | 2, -2 | | 3, -2 | | 3, -3 | | 3, -2 | | 4, -3 | | 3, -3 | | Survival | 95.31 |
| 11 | 2, -2 | | 1, -2 | | 3, -3 | | 1, -2 | | 2, -3 | | 2, -3 | | 3, -4 | | Survival | 92.23 |
| 12 | 2, -1 | | 2, 0 | | 3, -2 | | 1, 0 | | 2, -1 | | 3, -3 | | 5, -3 | | Survival | 94.25 |
| 13 | 0, -2 | | 2, -1 | | 1, -1 | | 2, 0 | | 3, -1 | | 3, -2 | | 4, -3 | | Survival | 92.96 |
| 14 | 1, 0 | | 1, 0 | | 2, 0 | | 3, -1 | | 3, -2 | | 4, -4 | | 3, -4 | | Survival | 93.68 |
| 15 | 0, 0 | | 1, -2 | | 1, 0 | | 2, -2 | | 3, -1 | | 4, -3 | | 4, -4 | | Survival | 94.76 |
| 16 | 1, -2 | | 2, -1 | | 1, -2 | | 2, -1 | | 3, -2 | | 2, -2 | | 3, -4 | | Survival | 97.60 |
| 17 | 2, -2 | | 1, -1 | | 2, -1 | | 3, -1 | | 4, -2 | | 3, -3 | | 4, -3 | | Survival | 94.25 |
| 18 | 1, -1 | | 2, 0 | | 2, -2 | | 2, -2 | | 3, -3 | | 3, -2 | | 4, -3 | | Survival | 93.12 |
| 19 | 0, -1 | | 1, -1 | | 1, 0 | | 2, -2 | | 3, -2 | | 2, -3 | | 3, -3 | | Survival | 94.52 |
| 20 | 1, -1 | | 2, 0 | | 1, -2 | | 2, -2 | | 3, -3 | | 2, -3 | | 3, -2 | | Survival | 93.75 |
| 21 | 1, 0 | | 1, -2 | | 2, -1 | | 3, -2 | | 2, -3 | | 3, -2 | | 3, -3 | | Survival | 95.35 |
| 22 | 2, -1 | | 3, -2 | | 4, -3 | | 3, -4 | | 3, -2 | | 4, -3 | | 3, -2 | | Survival | 96.33 |
| 23 | 0, -1 | | 1, 0 | | 2, -2 | | 2, -1 | | 3, -3 | | 3, -2 | | 3, -3 | | Survival | 91.02 |
| 24 | 2, 0 | | 2, -2 | | 2, -1 | | 3, -2 | | 2, -3 | | 3, -4 | | 3, -3 | | Survival | 91.87 |
| 25 | 2, 0 | | 1, -1 | | 0, -2 | | 2, -1 | | 3, -3 | | 3, -2 | | 3, -3 | | Survival | 92.30 |
| 26 | 1, 0 | | 2, -2 | | 1, -3 | | 2, -2 | | 3, -2 | | 3, -3 | | 3, -2 | | Survival | 90.27 |
| 27 | 2, -1 | | 1, 0 | | 2, -2 | | 3, -3 | | 3, -2 | | 2, -2 | | 4, -2 | | Survival | 94.97 |
| 28 | 1, -1 | | 2, -2 | | 1, -2 | | 3, -3 | | 3, -2 | | 2, -3 | | 3, -3 | | Survival | 93.45 |
| 29 | 1, -1 | | 0, -1 | | 2, -3 | | 3, -2 | | 4, -3 | | 3, -4 | | 3, -3 | | Survival | 94.58 |
| 30 | 2, 0 | | 1, 0 | | 2, -2 | | 2, -3 | | 3, -2 | | 4, -3 | | 3, -2 | | Survival | 94.48 |
| 31 | 1, -1 | | 2, -2 | | 2, -1 | | 3, -2 | | 2, -3 | | 3, -3 | | 4, -3 | | Survival | 91.01 |
| 32 | 0, -1 | | 2, -2 | | 4, -2 | | 4, -4 | | 3, -6 | | 4, -5 | | 4, -5 | | Necrosis | 79.45 |
| 33 | 2, 0 | | 2, -1 | | 5, -3 | | 5, -4 | | 5, -5 | | 5, -5 | | 5, -6 | | Necrosis | 70.45 |
| 34 | 1, 0 | | 2, -3 | | 4, -2 | | 4, -3 | | 4, -4 | | 5, -4 | | 4, -4 | | Necrosis | 77.65 |
| 35 | 0, -2 | | 1, 0 | | 4, -3 | | 5, -5 | | 5, -3 | | 5, -6 | | 4, -6 | | Necrosis | 78.94 |
| 36 | 1, -1 | | 3, -2 | | 5, -3 | | 4, -6 | | 5, -4 | | 4, -6 | | 6, -7 | | Necrosis | 70.74 |
| 37 | 2, 0 | | 2, -1 | | 5, -3 | | 5, -4 | | 4, -3 | | 5, -5 | | 5, -5 | | Necrosis | 78.69 |
| 38 | 2, -2 | | 2, -1 | | 4, -3 | | 5, -4 | | 4, -4 | | 4, -5 | | 6, -7 | | Necrosis | 77.09 |

**Patients with treatment under the guidance of s**kin barrier monitoring technology** diagnosis after RPSFs surgeries (Group A)**

**Patients with treatment under the guidance of s**kin barrier monitoring technology** diagnosis after RPSFs surgeries (Group A)**

| No. | D1 | | D2 | | D3 | | D4 | | D5 | | D6 | | D7 | |  |  |
| --- | --- | --- | --- | --- | --- | --- | --- | --- | --- | --- | --- | --- | --- | --- | --- | --- |
|  | RT | RS | RT | RS | RT | RS | RT | RS | RT | RS | RT | RS | RT | RS | Outcome | SR (%) |
| 39 | 1, -1 | | 0, -2 | | 5, -2 | | 4, -5 | | 5, -3 | | 5, -4 | | 7, -6 | | Necrosis | 76.45 |
| 40 | 1, -2 | | 2, -3 | | 4, -4 | | 5, -4 | | 4, -4 | | 6, -5 | | 6, -5 | | Necrosis | 83.48 |
| 41 | 0, -2 | | 1, -1 | | 4, -3 | | 5, -6 | | 6, -5 | | 6, -6 | | 6, -5 | | Necrosis | 68.10 |
| 42 | 1, -1 | | 2, -2 | | 4, -3 | | 5, -5 | | 4, -5 | | 5, -4 | | 7, -6 | | Necrosis | 63.51 |
| 43 | 0, -1 | | 2, -1 | | 4, -4 | | 4, -3 | | 5, -4 | | 6, -5 | | 7, -7 | | Necrosis | 79.67 |
| 44 | 1, -2 | | 0, -1 | | 3, -2 | | 3, -3 | | 4, -3 | | 5, -4 | | 8, -7 | | Necrosis | 73.20 |
| 45 | 0, -1 | | 0, -1 | | 4, -3 | | 4, -4 | | 5, -4 | | 6, -5 | | 7, -6 | | Necrosis | 77.75 |

RPSFs: Random-pattern skin flaps, SR: Survival rate, RT: Relative transepidermal water loss, RS: Rrelative stratum corneum hydration.
