# Supplementary material for: Innovative research on predicting skin flaps necrosis through quantification of the USP4/TNFAIP2 regulated ‘M1 macrophages-Tregs axis’ via skin barrier monitoring technology
Source: Burns Trauma. 2026 Feb 27;14:tkag016. doi: 10.1093/burnst/tkag016 (PMC13602267; doi:10.1093/burnst/tkag016)
Supplement: Supplementary file 1 [file supplementary_material_tkag016.zip › supplementary Table3.2.docx]

**Table3.2**

**Patients without treatment under the guidance of s**kin barrier monitoring technology** diagnosis after RPSFs surgeries (Group B)**

| No. | D1 | | D2 | | D3 | | D4 | | D5 | | D6 | | D7 | |  |  |
| --- | --- | --- | --- | --- | --- | --- | --- | --- | --- | --- | --- | --- | --- | --- | --- | --- |
|  | RT | RS | RT | RS | RT | RS | RT | RS | RT | RS | RT | RS | RT | RS | Outcome | SR (%) |
| 1 | 0, -1 | | 2, -2 | | 1, -1 | | 2, -2 | | 2, -2 | | 3, -2 | | 4, -2 | | Survival | 91.28 |
| 2 | 1, -1 | | 0, -2 | | 1, -1 | | 2, -2 | | 2, -3 | | 3, -2 | | 3, -3 | | Survival | 89.16 |
| 3 | 1, 0 | | 1, -1 | | 2, -2 | | 1, -2 | | 3, -3 | | 3, -3 | | 3, -4 | | Survival | 96.09 |
| 4 | 0, 0 | | 1, -2 | | 2, -1 | | 1, -2 | | 2, -2 | | 4, -2 | | 3, -3 | | Survival | 90.16 |
| 5 | 1, 0 | | 0, -1 | | 1, 0 | | 2, -1 | | 3, -3 | | 3, -2 | | 3, -2 | | Survival | 91.25 |
| 6 | 2, -2 | | 1, -1 | | 1, -2 | | 1, -2 | | 2, -2 | | 4, -2 | | 3, -3 | | Survival | 91.82 |
| 7 | 1, -1 | | 2, 0 | | 2, -1 | | 1, -3 | | 2, -3 | | 2, -1 | | 4, -2 | | Survival | 90.35 |
| 8 | 2, 0 | | 3, -1 | | 4, -3 | | 3, -3 | | 3, -2 | | 4, -3 | | 3, -3 | | Survival | 93.97 |
| 9 | 2, -1 | | 1, -1 | | 1, -1 | | 2, -3 | | 3, -1 | | 3, -3 | | 3, -3 | | Survival | 91.59 |
| 10 | 1, -2 | | 2, -2 | | 2, -1 | | 2, -3 | | 4, -2 | | 3, -3 | | 4, -2 | | Survival | 94.79 |
| 11 | 1, -1 | | 0, 0 | | 2, -2 | | 2, -2 | | 3, -3 | | 4, -4 | | 4, -4 | | Survival | 93.86 |
| 12 | 1, 0 | | 1, -1 | | 1, -2 | | 2, -1 | | 3, -1 | | 3, -3 | | 3, -3 | | Survival | 89.08 |
| 13 | 2, -1 | | 3, -2 | | 4, -3 | | 1, -2 | | 3, -1 | | 2, -3 | | 5, -4 | | Survival | 96.19 |
| 14 | 2, -2 | | 2, -1 | | 1, -1 | | 1, 0 | | 2, -2 | | 3, -3 | | 4, -2 | | Survival | 97.37 |
| 15 | 0, -2 | | 1, 0 | | 2, -1 | | 3, -3 | | 3, -1 | | 2, -2 | | 5, -4 | | Survival | 90.49 |
| 16 | 1, -1 | | 1, -2 | | 2, -1 | | 3, -2 | | 2, -2 | | 3, -3 | | 4, -3 | | Survival | 89.44 |
| 17 | 1, 0 | | 2, -2 | | 3, -2 | | 2, -2 | | 4, -2 | | 2, -3 | | 3, -4 | | Survival | 94.85 |
| 18 | 0, -2 | | 1, -1 | | 1, -1 | | 3, -1 | | 3, -3 | | 4, -2 | | 4, -2 | | Survival | 95.85 |
| 19 | 1, -1 | | 0, -2 | | 1, 0 | | 2, -2 | | 4, -3 | | 2, -2 | | 4, -4 | | Survival | 88.00 |
| 20 | 2, -2 | | 3, 0 | | 4, -2 | | 2, -1 | | 2, -4 | | 2, -3 | | 3, -2 | | Survival | 93.21 |
| 21 | 2, -1 | | 1, -2 | | 2, -2 | | 3, -1 | | 4, -2 | | 3, -3 | | 4, -4 | | Survival | 91.22 |
| 22 | 1, 0 | | 2, -1 | | 3, -2 | | 3, -2 | | 4, -3 | | 3, -1 | | 3, -2 | | Survival | 90.47 |
| 23 | 2, -1 | | 0, -2 | | 2, -1 | | 1, -2 | | 3, -3 | | 2, -3 | | 4, -2 | | Survival | 91.95 |
| 24 | 0, -2 | | 1, -1 | | 2, 0 | | 3, -3 | | 3, -2 | | 2, -3 | | 4, -3 | | Survival | 90.43 |
| 25 | 1, -1 | | 1, 0 | | 2, -1 | | 2, -2 | | 3, -3 | | 3, -3 | | 2, -3 | | Survival | 89.54 |
| 26 | 1, 0 | | 1, -2 | | 2, -2 | | 3, -2 | | 3, -3 | | 4, -2 | | 3, -2 | | Survival | 95.96 |
| 27 | 2, 0 | | 2, -2 | | 3, -3 | | 3, -3 | | 2, -2 | | 4, -2 | | 3, -3 | | Survival | 90.82 |
| 28 | 0, -1 | | 1, -2 | | 2, -1 | | 3, -2 | | 4, -3 | | 4, -3 | | 3, -2 | | Survival | 91.72 |
| 29 | 1, -2 | | 2, 0 | | 1, -1 | | 2, -2 | | 3, -3 | | 3, -2 | | 4, -3 | | Survival | 90.74 |
| 30 | 2, 0 | | 1, -3 | | 2, -1 | | 3, -3 | | 4, -2 | | 3, -4 | | 4, -3 | | Survival | 92.04 |
| 31 | 1, -1 | | 2, -2 | | 3, -3 | | 2, -2 | | 3, -4 | | 2, -3 | | 4, -3 | | Survival | 89.01 |
| 32 | 1, 0 | | 0, -2 | | 2, -1 | | 3, -3 | | 3, -2 | | 4, -3 | | 3, -2 | | Survival | 96.87 |
| 33 | 0, -1 | | 2, -2 | | 3, -2 | | 2, -2 | | 3, -3 | | 2, -1 | | 3, -3 | | Survival | 93.39 |
| 34 | 1, 0 | | 1, -1 | | 4, -4 | | 6, -5 | | 6, -6 | | 5, -8 | | 7, -8 | | Necrosis | 72.74 |
| 35 | 2, -1 | | 2, -1 | | 4, -4 | | 5, -5 | | 8, -7 | | 10, -9 | | 10, -12 | | Necrosis | 58.71 |
| 36 | 0, -1 | | 1, -2 | | 5, -2 | | 6, -3 | | 7, -5 | | 7, -6 | | 9, -7 | | Necrosis | 77.20 |
| 37 | 1, -2 | | 2, -1 | | 5, -4 | | 5, -3 | | 6, -5 | | 8, -7 | | 8, -9 | | Necrosis | 65.51 |
| 38 | 1, 0 | | 2, -2 | | 4, -2 | | 5, -4 | | 7, -6 | | 9, -8 | | 13, -10 | | Necrosis | 47.87 |

**Patients without treatment under the guidance of s**kin barrier monitoring technology** diagnosis after RPSFs surgeries (Group B)**

| No. | D1 | | D2 | | D3 | | D4 | | D5 | | D6 | | D7 | |  |  |
| --- | --- | --- | --- | --- | --- | --- | --- | --- | --- | --- | --- | --- | --- | --- | --- | --- |
|  | RT | RS | RT | RS | RT | RS | RT | RS | RT | RS | RT | RS | RT | RS | Outcome | SR (%) |
| 39 | 2, -1 | | 3, -2 | | 5, -4 | | 4, -5 | | 6, -6 | | 7, -7 | | 7, -8 | | Necrosis | 69.49 |
| 40 | 1, -2 | | 2, -3 | | 4, -3 | | 5, -3 | | 7, -5 | | 6, -5 | | 9, -6 | | Necrosis | 51.87 |
| 41 | 1, 0 | | 2, -2 | | 4, -3 | | 4, -3 | | 5, -4 | | 6, -4 | | 7, -6 | | Necrosis | 63.60 |
| 42 | 0, -2 | | 3, -1 | | 5, -3 | | 6, -5 | | 5, -6 | | 7, -8 | | 9, -8 | | Necrosis | 68.49 |
| 43 | 2, -2 | | 1, 0 | | 4, -4 | | 4, -5 | | 6, -7 | | 8, -9 | | 12, -10 | | Necrosis | 53.88 |
| 44 | 2, -2 | | 1, -2 | | 4, -3 | | 5, -5 | | 8, -7 | | 10, -9 | | 14, -10 | | Necrosis | 50.16 |
| 45 | 0, -1 | | 2, -1 | | 3, -4 | | 7, -6 | | 6, -5 | | 6, -5 | | 6, -7 | | Necrosis | 73.88 |

RPSFs: Random-pattern skin flaps, SR: Survival rate, RT: Relative transepidermal water loss, RS: Rrelative stratum corneum hydration.
