# Supplementary material for: Innovative research on predicting skin flaps necrosis through quantification of the USP4/TNFAIP2 regulated ‘M1 macrophages-Tregs axis’ via skin barrier monitoring technology
Source: Burns Trauma. 2026 Feb 27;14:tkag016. doi: 10.1093/burnst/tkag016 (PMC13602267; doi:10.1093/burnst/tkag016)
Supplement: Supplementary file 1 [file supplementary_material_tkag016.zip › supplementary Table4.docx]

****Table 4. Comparison of necrosis incidence between Group A and Group B****

| Group | Necrosis | Survival | Total |
| --- | --- | --- | --- |
| Group A | 14 | 31 | 45 |
| Group B | 12 | 33 | 45 |
| Total | **26** | **64** | **90** |

The chi-square test revealed no significant difference in the number of necrosis cases between the groups (χ² = 0.2163, p = 0.6418).
