# Supplementary material for: Innovative research on predicting skin flaps necrosis through quantification of the USP4/TNFAIP2 regulated ‘M1 macrophages-Tregs axis’ via skin barrier monitoring technology
Source: Burns Trauma. 2026 Feb 27;14:tkag016. doi: 10.1093/burnst/tkag016 (PMC13602267; doi:10.1093/burnst/tkag016)
Supplement: Supplementary file 1 [file supplementary_material_tkag016.zip › supplementary Table5.1.docx]

****Table 5.1 Diagnostic performance of RT (> 3.5) for necrosis prediction****

| **RT Test Result** | **Necrosis (Positive)** | **Survival (Negative)** | **Total** |
| --- | --- | --- | --- |
| **Positive (RT >3.5)** | **11**(TP) | **3** (FP) | **14** |
| **Negative (RT < 3.5)** | **1** (FN) | **30** (TN) | **31** |
| **Total** | **12** | 33 | **45** |

RT: Relative transepidermal water loss, TP: True positive, FP: False positive, FN: False n**egative, TN: True** n**egative.** The **sensitivity** and **specificity** of RT (>3.5) for predicting necrosis were **91.67**% (64.06% – 98.52%) and **90.91**% (76.93% – 96.94%), respectively.
